# Associations Between Occupational Exposures and Cough Subclasses Among Middle‐Aged Australians

**DOI:** 10.1111/resp.70040

**Published:** 2025-04-02

**Authors:** Jingwen Zhang, Jennifer L. Perret, Dinh S. Bui, Sheikh M. Alif, Michael J. Abramson, Anne B. Chang, Hans Kromhout, Garun S. Hamilton, Paul S. Thomas, Bircan Erbas, Bruce R. Thompson, Melanie C. Matheson, E. Haydn Walters, Caroline J. Lodge, Shyamali C. Dharmage

**Affiliations:** ^1^ Allergy and Lung Health Unit, Centre for Epidemiology and Biostatistics Melbourne School of Population and Global Health, The University of Melbourne Melbourne Victoria Australia; ^2^ Institute for Breathing and Sleep (IBAS) Victoria Australia; ^3^ School of Public Health and Preventive Medicine Monash University Melbourne Victoria Australia; ^4^ Institute of Health and Wellbeing Federation University Australia Berwick Victoria Australia; ^5^ Melbourne School of Health Science, The University of Melbourne Melbourne Victoria Australia; ^6^ Australian Centre for Health Services Innovation Queensland University of Technology Brisbane Queensland Australia; ^7^ Child Health Division Menzies School of Health Research, Charles Darwin University Northwest Territories Australia; ^8^ Institute for Risk Assessment Sciences, Utrecht University Utrecht the Netherlands; ^9^ Department of Lung, Sleep, Allergy & Immunology Monash Health Clayton Victoria Australia; ^10^ School of Clinical Sciences Monash University Clayton Victoria Australia; ^11^ Prince of Wales' Clinical School, Faculty of Medicine UNSW and Respiratory Medicine Sydney New South Wales Australia; ^12^ Prince of Wales' Hospital Randwick New South Wales Australia; ^13^ School of Psychology and Public Health La Trobe University Melbourne Victoria Australia; ^14^ Population Health Solutions Telstra Health Melbourne Victoria Australia; ^15^ School of Medicine University of Tasmania Hobart Tasmania Australia

**Keywords:** allergy, COPD, cough, Environmental & Occupational Health and Epidemiology

## Abstract

**Background and Objective:**

The evidence around occupation‐related chronic cough is conflicting and current definitions of chronic cough cannot capture its heterogeneity. Using our recently characterised novel cough subclasses, we aimed to identify subclass‐specific occupational risks.

**Methods:**

Using data from the Tasmanian Longitudinal Health Study (TAHS), occupational exposures up to age 53 years were coded using the ALOHA+ Job Exposure Matrix, into ever‐exposure (no, only‐low, ever‐high) and cumulative exposure. People belonging to six previously identified cough subclasses among 2213 current coughers at age 53 years were compared to non‐coughers (*n* = 1396). Associations with occupational exposures were assessed using multinomial logistic regression for these cough subclasses and logistic regression for standard definitions (chronic cough, chronic phlegm, and chronic bronchitis) after adjusting for potential confounders.

**Results:**

Biological dust was associated with “cough with allergies” (cumulative: adjusted multinomial odds ratio [aMOR] = 1.06, 95% CI: 1.02–1.10, per 10 exposure‐year increase). Aromatic solvents were associated with “chronic dry cough” (cumulative: aMOR = 1.15, 95% CI: 1.02–1.29). Other solvents were associated with “chronic productive cough” (ever‐high: aMOR = 2.81, 95% CI: 1.26–6.2); “intermittent productive cough” (cumulative: aMOR = 1.06, 95% CI: 0.98–1.16), chronic bronchitis (ever‐high: aOR = 2.48, 95% CI: 1.01–6.06); and chronic phlegm (ever‐high: aOR = 2.26, 95% CI: 1.14–4.51). Herbicides (cumulative) were also associated with “intermittent productive cough” (aOR = 1.09, 95% CI: 1.00–1.77) and chronic phlegm (aOR = 1.07, 95% CI: 1.00–1.15).

**Conclusion:**

Novel cough subclasses had distinct associations with specific occupational exposures, suggesting different pathophysiology. Aromatic solvents were associated with dry cough; biological dust with allergic cough; herbicides and other solvents with productive cough. Using novel cough subclasses was superior to standard definitions in uncovering these associations.

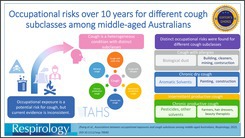

## Graphical abstract

This study explored novel cough subclasses and their associations with occupational exposures. Findings revealed new links between specific exposures and cough types, contributing to the literature by identifying distinct occupational risks for chronic dry, chronic productive, and allergic coughs. These outcomes have clinical impact by emphasizing the importance of detailed occupational history in cough assessment.

**See related** editorial

## 1 Introduction

Chronic cough of 8 weeks or greater duration affects up to 10% of the adult population globally, with patients in their sixth decade of life most commonly presenting to healthcare providers [1, 2]. Recent studies have found chronic cough to be associated with increased mortality, independent of smoking and lung function [3, 4]. Cough is a common symptom of many chronic respiratory diseases and is a heterogeneous condition [5]. To address this heterogeneity, which is currently poorly understood, our recent work used latent class analysis, an unsupervised data‐driven method, to identify six novel cough subclasses in a general population, each with distinct clinical characteristics from childhood to middle age [6]. This provides the opportunity to investigate risk factors for different cough subclasses and to inform subclass‐specific prevention and management strategies. Uniquely, these subclasses were derived from community‐based data and not from hospital clinics.

Occupational exposures are important potential modifiable risk factors for many respiratory diseases, including cough, and have been estimated to contribute 13% to the burden of chronic bronchitis [7]. However, when assessing cough patients in clinical practice, occupational causes of cough were not addressed in detail [8], possibly due to the inconsistency in the literature on associations between occupational exposures and chronic cough [9]. Studies conducted to date have used one or more standard clinical definitions of cough (i.e., chronic cough [CC], chronic phlegm [CP], and chronic bronchitis [CB]), but results for the same definition are still inconsistent.

For example, one study found vapours, gases, dust, or fumes (VGDF) to be associated with CB in a historical (1976–1983) cohort, but only for smokers in a contemporary (2003–2017) cohort [10]. Two other studies found associations between dust and CB, but only among male participants [11, 12]. However, no associations and/or interactions by sex/smoking were found in another two studies [13, 14]. Besides, the occupational risks of the three standard definitions (i.e., CC, CP, CB) were usually similar within a single study but different across studies [13, 14]. Such inconsistency suggests that different cough subclasses and their heterogeneous associations with different occupational exposures cannot be fully identified by using the standard cough definitions.

Therefore, we aimed to investigate the associations between occupational exposures and our new classification of cough subclasses [6] in a general middle‐aged population. We also investigated relationships with cough using standard chronic cough definitions (undifferentiated CC, chronic phlegm, and chronic bronchitis) to compare their performance with the cough subclasses and results from previous studies.

## 2 Methods

### 2.1 Study Design and Population

We used data from the Tasmanian Longitudinal Health Study (TAHS) cohort. Details about the study design were published previously [15]. Briefly, almost all (99%) school children born in Tasmania, Australia in 1961 were recruited when they were 7 years old (*n* = 8583); those enrolled underwent a clinical examination, and their parents completed a respiratory health questionnaire. From 2012 to 2016 (mean age of 53 years, the 6th‐decade follow‐up), all surviving participants were traced and invited to the study (*n* = 6128), of whom 3609 (59%) returned a postal questionnaire and were included in this analysis (Figure S1). They were also asked to complete a lifetime work history calendar. The study was approved by Human Ethics Review Committees at all participating institutions, and written informed consent was obtained from all participants.

### 2.2 Data Collection and Definition of Variables

#### 2.2.1 Occupational Exposures

The work history calendar completed by participants recorded details about all jobs until the 6th‐decade follow‐up at age 53 years, including job titles, industry descriptions, employer descriptions, and the year work started and ended. Job titles were coded using the International Standard Classification of Occupations (ISCO‐88) and were then linked to ALOHA+ Job Exposure Matrix (JEM), a modified population‐based JEM originally developed for chronic bronchitis [16, 17]. A job exposure matrix (JEM) comprises a list of levels of likely exposure to various harmful or potentially harmful agents for specific occupations, based on self‐reported job titles and duration of the job. Its use in occupational epidemiological studies enables researchers to quantify the exposure of potential health hazards to individuals while in the workplace. JEMs are cost‐effective tools widely used in large‐scale population‐based studies when documented individual‐level occupational exposures are unavailable, which is usually the case [18]. Using ALOHA+ JEM, coded job titles were assigned no, low, or high exposure levels (0, 1, 2) for exposures including dust/gases (biological dust, mineral dust, gases/fumes); pesticides (fungicides, herbicides, insecticides); solvents (aromatic, chlorinated, other solvents); and metals. Where participants had two different jobs at the same time, each job was calculated as half of the exposure time and then added up [18, 19].

Two variables, ever‐exposure and cumulative exposure, were calculated for each agent based on the ALOHA + JEM. Ever‐exposure was defined by the maximum exposure of any agent over the participants' entire working career up until age 53 years and was categorised into no (reference level), only‐low, or ever‐high exposures. Cumulative unit‐year was a semi‐quantitative variable defined as the years worked under a job title multiplied by the intensity of any exposure related to the job title (1 for low exposure and 4 for high exposure) [19] and was scaled to cumulative exposure per 10 years for analysis. For each participant, the cumulative exposure was the sum of exposures over the entire job history, calculated for each agent [18].

#### 2.2.2 Cough Subclasses and Standard Cough Definitions

Nine self‐reported cough and/or phlegm symptoms collected via questionnaires were used to derive the cough subclasses (Table S1). Details on the derivation of the cough subclass have been described previously and summarised in Table S1 [6]. Briefly, 2213 of the 3609 participants were labelled as “current coughers” as they reported a cough symptom at age 53 years and were further classified into six cough subclasses using latent class analysis (namely, “minimal cough”, “cough with colds only”, “cough with allergies”, “intermittent productive cough”, “chronic dry cough”, and “chronic productive cough”). The remaining 1396 participants who did not report any cough symptoms at age 53 years were labelled as “non‐coughers” and made up the reference group for all comparisons in the analyses. The cross‐sectional and longitudinal clinical features of these cough subclasses were summarised in Table S2. Standard definitions of cough included chronic cough (CC, cough ≥ 3 months); chronic phlegm (CP, phlegm ≥ 3 months); and chronic bronchitis (CB, cough with phlegm for ≥ 3 months in 2 consecutive years) (Table S3).

### 2.3 Statistical Analysis

Characteristics of the participants were tabulated according to their cough subclasses and compared with the reference group (non‐coughers), using χ^2^ tests or independent t‐tests. Correlations between the occupational exposures were assessed using Spearman's correlation coefficients (ρ). Multinomial logistic regressions were conducted to investigate the associations between each of the agents (as ever‐exposure, and cumulative) and the cough subclasses. These analyses were adjusted for a minimum set of confounders that included sex, education, smoking, pack‐years, and asthma in childhood and adulthood (main analysis). Confounders were identified based on literature reviews and a directed acyclic graph (DAG) [20], and were defined in Table S3. To assess potential confounding introduced by correlations between the occupational exposures, sensitivity analyses were conducted by: (i) comparing to a common control group consisting of participants not exposed to any of the agents (unexposed analysis); and (ii) mutually adjusting multiple correlated exposures (ρ ≥ 0.3, co‐exposure analysis). Additionally, analysis limited to participants exposed to a single agent was conducted if needed to confirm potential confounding (subgroup analysis).

Multivariable logistic regression models were conducted for the cough outcomes using standard definitions (CC, CP, and CB). Effect modification by sex and smoking on CC, CP, and CB was assessed by including interaction terms (i.e., exposure × sex, exposure × smoking) in the models and comparing models using likelihood ratio tests. Stratified estimates were reported if the p‐values for both the likelihood ratio test and the interaction term were less than 0.1. All statistical analyses were done in STATA, version 16 (Stata Corp, College Station, TX).

## 3 Results

### 3.1 Study Population

Of the 3609 participants, 3242 (89.8%) filled out the work history calendar. The demographic features of these 3242 participants were similar to those of the 367 participants who did not fill out the work history calendar, except that there were more smokers among the 367 participants (Table S4). Around a quarter of the 3242 participants had university or higher education, 49% were males, 17% were current smokers, 33% were obese, and 15% had current asthma at age 53 years. There were 1242 (38%) “non‐coughers” making up the reference group for all comparisons, while the remaining 2000 participants were assigned to one of six cough subclasses (Table 1).

**TABLE 1:** Demographic characteristics of participants at age 53 years (*N* = 3242).

Demographic characteristics		Participants included for the occupational risk analysis (*N* = 3242)	
Male		1578 (49%)	
Age, years		53 ± 1	
Highest education	< Grade 12	1072 (33%)	
	Grade 12 or equivalent	1376 (43%)	
	University or higher	759 (24%)	
Smoking	Never	1432 (44%)	Pack‐years: median, IQR (*n*)
	Past	1246 (39%)	8, 17 (1110)
	Current	557 (17%)	24, 23 (527)
BMI (kg/m^2^)		29 ± 6	
Obesity (BMI > 30 kg/m^2^)		1046 (33%)	
Adulthood asthma		432 (13%)	
Childhood asthma		476 (15%)	
Chronic cough		337 (10%)	
Chronic phlegm		189 (6%)	
Chronic bronchitis		96 (3%)	
Cough subclasses at age 53 years		*N* (%)	
Non‐coughers (reference group)		1242 (38%)	
Minimal cough		181 (6%)	
Cough with colds only		1069 (33%)	
Cough with allergies		281 (9%)	
Intermittent productive cough		195 (6%)	
Chronic dry cough		135 (4%)	
Chronic productive cough		139 (4%)	

*Note*: Numbers are presented as crude numbers (%) for categorical variables; mean ± standard deviation for continuous variables; median, IQR (*n*, number of participants with values > 0) for pack‐years (missing data for 166 participants).

Abbreviations: BMI, body mass index in kg/m^2^, obesity defined as BMI > 30 kg/m^2^; IQR, inter‐quartile range.

The prevalence of occupational exposures is presented in Table S5. The most common exposures were gases and fumes (72%), biological dust (56%), mineral dust (49%), and other solvents (40%). These relatively high exposure rates reflect the occupational profile of Tasmania, which has a higher proportion of workers in agriculture and other rural industries compared to other parts of Australia. Distributions of occupational exposures across the cough subclasses are shown in Tables S6 and S7.

### 3.2 Occupational Exposures and Cough Outcomes

Associations between occupational exposures and the six novel cough subclasses were seen when measured as ever‐exposure (Table 2) or cumulative exposure (Table 3) and were summarised in Tables 4 and 5. Ever‐exposure to dust/gases (only‐low, adjusted multinomial odds ration [aMOR] = 1.49, 95% CI: 1.04–2.15; ever‐high, aMOR = 1.71, 95% CI: 1.14–2.56), cumulative exposures to biological dust (aMOR = 1.06, 95% CI: 1.02–1.10), and to pesticides (e.g., herbicides aMOR = 1.09, 95% CI: 1.02–1.17) were all associated with the “cough with allergies” subclass. There was also some evidence of the association between ever‐high exposure to herbicides and the “cough with allergies” subclass (aMOR = 1.56, 95% CI: 0.98–2.46).

**TABLE 2:** Adjusted associations between occupational exposures (ever‐exposure) and cough subclasses at age 53 years.

Ever occupational exposures		Cough subclasses at age 53 years (*N* = 3022)					
		Minimal cough (*n* = 173)	Cough with colds only (*n* = 1001)	Cough with allergies (*n* = 264)	Intermittent productive cough (*n* = 178)	Chronic dry cough (*n* = 122)	Chronic productive cough (*n* = 129)
		aMOR (95% CI)					
Biological dust	L	1.01 (0.70–1.46)	1.08 (0.89–1.31)	1.63 (1.17–2.20)	1.10 (0.76–1.59)	0.78 (0.49–1.22)	0.91 (0.58–1.43)
	H	0.84 (0.53–1.33)	0.88 (0.69–1.13)	1.75 (1.17–2.60)	0.87 (0.55–1.37)	1.19 (0.72–1.97)	0.98 (0.58–1.65)
Mineral dust	L	1.24 (0.82–1.89)	1.13 (0.89–1.42)	1.27 (0.88–1.84)	1.34 (0.89–2.02)	1.28 (0.78–2.07)	1.25 (0.76–2.04)
	H	1.06 (0.68–1.64)	1.10 (0.87–1.39)	1.61 (1.11–2.34)	0.86 (0.55–1.36)	0.99 (0.58–1.68)	0.99 (0.58–1.68)
Gases and fumes	L	1.19 (0.80–1.78)	1.14 (0.93–1.41)	1.60 (1.14–2.25)	1.55 (1.02–2.36)	0.95 (0.60–1.49)	1.28 (0.78–2.11)
	H	1.29 (0.80–2.07)	1.17 (0.91–1.51)	1.78 (1.16–2.72)	1.17 (0.71–1.93)	0.99 (0.57–1.72)	1.21 (0.68–2.17)
Herbicides	L	0.63 (0.27–1.51)	1.03 (0.70–1.53)	1.24 (0.67–2.28)	0.82 (0.39–1.76)	0.69 (0.26–1.81)	1.22 (0.56–2.65)
	H	0.95 (0.54–1.68)	1.01 (0.75–1.38)	1.56 (0.98–2.46)	1.39 (0.85–2.28)	0.98 (0.50–1.82)	1.12 (0.59–2.12)
Insecticides	L	0.73 (0.2–1.90)	0.62 (0.37–1.04)	1.09 (0.53–2.23)	0.64 (0.24–1.70)	0.76 (0.26–2.23)	1.05 (0.40–2.71)
	H	0.80 (0.46–1.40)	1.09 (0.83–1.44)	1.44 (0.94–2.21)	1.19 (0.74–1.92)	0.76 (0.39–1.48)	0.88 (0.48–1.63)
Fungicides	L	0.97 (0.44–2.13)	0.64 (0.39–1.04)	0.96 (0.45–2.02)	0.66 (0.27–1.60)	1.45 (0.64–3.26)	0.61 (0.20–1.82)
	H	0.75 (0.43–1.32)	1.04 (0.79–1.37)	1.44 (0.94–2.18)	1.12 (0.69–1.79)	0.83 (0.43–1.58)	1.02 (0.57–1.81)
Aromatic solvents	L	0.94 (0.62–1.44)	1.20 (0.96–1.50)	1.39 (0.97–2.01)	0.88 (0.57–1.33)	1.26 (0.77–2.05)	0.92 (0.55–1.54)
	H	0.69 (0.20–2.41)	0.77 (0.39–1.51)	0.27 (0.04–2.07)	0.54 (0.15–1.92)	2.04 (0.70–5.91)	2.29 (0.87–6.02)
Chlorinated solvents	L	0.94 (0.53–1.66)	1.09 (0.81–1.46)	0.71 (0.41–1.23)	0.69 (0.38–1.27)	1.26 (0.69–2.30)	1.33 (0.73–2.42)
	H	1.42 (0.81–2.48)	1.26 (0.91–1.73)	0.96 (0.52–1.77)	0.81 (0.44–1.51)	0.87 (0.39–1.93)	0.96 (0.45–2.06)
Other solvents	L	1.13 (0.80–1.59)	1.15 (0.96–1.38)	1.20 (0.89–1.62)	1.01 (0.71–1.44)	0.94 (0.62–1.43)	1.01 (0.66–1.55)
	H	0.89 (0.34–2.35)	1.05 (0.64–1.75)	0.28 (0.07–1.19)	0.94 (0.38–2.37)	1.63 (0.64–4.13)	2.81 (1.26–6.24)
Metals		0.88 (0.51–1.53)	0.95 (0.71–1.27)	0.78 (0.45–1.33)	0.63 (0.36–1.09)	1.41 (0.79–2.53)	0.90 (0.48–1.68)
	H	1.33 (0.76–2.33)	1.26 (0.92–1.74)	1.05 (0.58–1.92)	0.65 (0.35–1.22)	0.93 (0.42–2.05)	0.85 (0.41–1.79)

*Note*: No exposure as the reference. All compared to the “non‐coughers” as the reference group.

Abbreviations: H, ever‐high; L, only‐low; MOR, adjusted multinomial odds ratio, adjusted for sex, education, smoking, pack‐years, childhood asthma and adulthood asthma at age 53 years.

**TABLE 3:** Adjusted associations between cumulative exposures and cough subclasses at age 53 years.

Cumulative occupational exposures	Cough subclasses at age 53 years (*N* = 2984)					
	Minimal cough (*n* = 170)	Cough with colds only (*n* = 991)	Cough with allergies (*n* = 263)	Intermittent productive cough (*n* = 174)	Chronic dry cough (*n* = 118)	Chronic productive cough (*n* = 127)
	aMOR for per 10 exposure unit‐year increase (95% CI)					
Biological dust	1.00 (0.95–1.05)	0.98 (0.96–1.02)	1.06 (1.02–1.10)	1.01 (0.96–1.06)	1.01 (0.95–1.08)	1.00 (0.93–1.07)
Mineral dust	1.00 (0.95–1.05)	1.00 (0.98–1.03)	1.03 (0.98–1.07)	0.97 (0.92–1.02)	0.96 (0.89–1.03)	0.99 (0.93–1.05)
Gases and fumes	1.03 (0.99–1.08)	1.00 (0.98–1.03)	1.01 (0.96–1.05)	1.01 (0.96–1.06)	1.03 (0.97–1.09)	1.01 (0.95–1.07)
Herbicides	1.02 (0.92–1.12)	0.98 (0.92–1.04)	1.09 (1.01–1.18)	1.09 (1.00–1.77)	0.98 (0.84–1.13)	1.03 (0.91–1.16)
Insecticides	0.98 (0.89–1.09)	0.99 (0.94–1.04)	1.09 (1.02–1.17)	1.04 (0.9–1.13)	0.93 (0.80–1.09)	0.98 (0.87–1.10)
Fungicides	0.97 (0.88–1.07)	0.98 (0.93–1.03)	1.08 (1.01–1.15)	1.03 (0.95–1.10)	1.00 (0.89–1.11)	1.00 (0.91–1.11)
Aromatic solvents	1.02 (0.89–1.17)	1.01 (0.93–1.09)	1.03 (0.90–1.18)	0.99 (0.86–1.14)	1.15 (1.02–1.29)	1.04 (0.90–1.20)
Chlorinated solvents	1.04 (0.98–1.11)	1.02 (0.99–1.06)	0.99 (0.92–1.07)	0.98 (0.91–1.06)	1.00 (0.91–1.10)	0.98 (0.88–1.08)
Other solvents	1.03 (0.94–1.13)	1.01 (0.95–1.06)	1.01 (0.92–1.10)	1.06 (0.98–1.16)	1.09 (0.99–1.20)	1.09 (0.99–1.20)
Metals	1.05 (0.99–1.11)	1.03 (0.99–1.06)	1.01 (0.94–1.08)	0.97 (0.90–1.05)	0.99 (0.90–1.09)	0.96 (0.87–1.06)

*Note*: Exposure unit: cumulative exposure unit year. All comparisons made with “non‐coughers” as the reference group.

Abbreviation: aMOR, adjusted multinomial odds ratio, adjusted for sex, education, smoking, pack‐years, childhood asthma and adulthood asthma at age 53 years.

**TABLE 4:** Summary of associations between occupational risks and cough outcomes at age 53 years.

Cough outcomes at mean age 53	Associated exposure	Notes and estimates (aMOR/aOR, 95% CI)
Novel cough subclasses		
Cough with allergies (*n* = 281)	Biological dust	Consistent associations with ever‐exposure (low‐level, aMOR = 1.63, 1.17–2.20; high‐level, aMOR = 1.75, 1.17–2.60) and cumulative exposure to biological dust (aMOR = 1.06, 1.02–1.10). The following agents all correlated with biological dust, and their associations were all attenuated in sensitivity analyses: mineral dust, gases and fumes, all pesticides, aromatic solvents.
Intermittent productive cough (*n* = 195)	Herbicides Other solvents	Consistent association with cumulative exposure to herbicides (aMOR = 1.09, 1.00–1.77). Borderline association with cumulative exposure to other solvents (aMOR = 1.06, 0.98–1.16) but became stronger in sensitivity analyses (aMOR = 1.11, 1.00–1.24). Association with low‐level ever‐exposure to gases and fumes was attenuated in subgroup analysis which excluded participants exposed to herbicides and other solvents.
Chronic dry cough (*n* = 135)	Aromatic solvents	Consistent association with cumulative exposure (aMOR = 1.15, 1.02–1.29) of aromatic solvents, similar association with other solvents was attenuated in sensitivity analyses. Consistently high point estimates for ever‐high exposure to aromatic solvents (i.e., aMOR ranges from 1.55 to 2.04), with wide 95% CIs in all analyses.
Chronic productive cough (*n* = 139)	Other solvents	Consistent associations with ever‐high exposure (aMOR = 2.81, 1.26–6.2), and cumulative to other solvents (aMOR = 1.09, 0.99–1.20). The association with aromatic solvents attenuated in all sensitivity analyses.
Standard cough definitions		
CB (*n* = 96)	Other solvents	Consistent associations with ever‐high exposure to other solvents (aOR = 2.48, 1.01–6.06). Sex interacted with cumulative exposure to other solvents (*p*‐for‐interaction = 0.095), with consistent association for females (aOR = 1.16, 1.02–1.33), but no association for males (aOR = 0.94, 0.78–1.13).
CP (*n* = 189)	Herbicides Other solvents	Consistent associations with both ever‐high exposure and cumulative exposures to herbicides and other solvents. Sex interacted with cumulative exposures to herbicides (*p*‐for‐interaction = 0.026), with associations with CP for males (aOR = 1.15, 1.06–1.24) but not for females (aOR = 0.86, 0.64–1.17). The associations and interaction by sex with dust/gases attenuated in all sensitivity analyses.

*Note*: Associations were considered “consistent” when associations remained unchanged (< 10%) or became stronger in the main analysis (Tables 2, 3, 4) and all sensitivity analyses (Tables S7–S14). The cough outcomes, “minimal cough”, “cough with colds only”, and the standard CC were not listed as there were no consistent associations found for them.

Abbreviations: aMOR, adjusted multinomial odds ratio, adjusted for sex, education, smoking, pack‐years, childhood asthma and adulthood asthma at age 53 years, and correlated agents if specified; CB, cough and phlegm ≥ 3 months and ≥ 2 years; CC, chronic cough, cough ≥ 3 months; CP, chronic phlegm, phlegm ≥ 3 months; Cumulative exposure unit‐year, cumulative exposure unit‐year, aMOR/aOR presented for evert per 10‐year increase.

**TABLE 5:** Summary of occupational exposures, their correlations, and associations with cough outcomes supported by consistent evidence.

Occupational exposures	Major findings (aMOR/aOR, 95% CI)
Biological dust	Consistent association with the “cough with allergies” subclass, with both ever‐exposures (only‐low, aMOR = 1.63, 1.17–2.20; ever‐high, aMOR = 1.75, 1.17–2.60) and cumulative exposure (aMOR = 1.06, 1.02–1.10). Correlated with mineral dust, gases and fumes, all pesticides, aromatic solvents, and other solvents.
Herbicides	Consistent association with the “intermittent productive cough” subclass (cumulative exposure, aMOR = 1.09, 1.00–1.77). Consistent associations with CP (ever‐high and cumulative exposure), interacted by sex (cumulative, aOR for males = 1.15, 1.06–1.24; aOR for females =0.86, 0.64–1.17; *p*‐for‐interaction = 0.026). Correlated with other insecticides, fungicides, and dust/gases. Over 99% of the participants exposed to herbicides were co‐exposed to dust/gases.
Aromatic solvents	Consistent association with the “chronic dry cough” subclass, with strong evidence for cumulative exposure (aMOR = 1.15, 1.02–1.29), and weak evidence for ever‐high exposure (i.e., wide 95% CIs but large point estimate, with aMOR ranges from 1.55 to 2.04). Correlated with other solvents.
Other solvents	Consistent association with the “intermittent productive cough” subclass, with moderate evidence for cumulative exposure (aMOR = 1.06, 0.98–1.16). Consistent association with the “chronic productive cough” subclass, with both ever‐high exposure (aMOR = 2.81, 1.26–6.2), and cumulative (aMOR = 1.09, 0.99–1.20). Consistent association with CB with ever‐high exposure (aOR = 2.48, 1.01–6.06) and cumulative interacted by sex (aOR for females = 1.16, 1.02–1.33; aOR for males = 0.94, 0.78–1.13; *p*‐for‐interaction = 0.095). Consistent association with CP with ever‐high exposure (aOR = 2.26, 1.14–4.51) and cumulative (aOR = 1.09, 0.99–1.20). Correlated with aromatic solvents, and dust/gases.

*Note*: Associations were considered “consistent” when associations remained unchanged (< 10%) or became stronger in the main analysis (Tables 3, 4, 5) and all sensitivity analyses (Tables S7–S14). Occupational exposures were not listed if there were no consistent associations (e.g., metals).

Abbreviations: aMOR, adjusted multinomial odds ratio, aOR, adjusted odds ratio, adjusted for sex, education, smoking, pack‐years, childhood asthma and adulthood asthma at age 53 years, and correlated agents if specified. CB, cough and phlegm ≥ 3 months and ≥ 2 years; CC, chronic cough, cough ≥ 3 months; CP, chronic phlegm, phlegm ≥ 3 months; Cumulative exposure unit‐year, cumulative exposure unit‐year, aMOR/aOR presented for evert per 10‐year increase.

There were associations between only‐low exposure to gases and fumes (aMOR = 1.55, 95% CI: 1.02–2.36), cumulative exposure to herbicides (aMOR = 1.09, 95% CI: 1.00–1.77), and the “intermittent productive cough” subclass.

Cumulative exposure to aromatic solvents was associated with the “chronic dry cough” subclass (aMOR = 1.15, 1.02–1.29). Ever‐high exposure to other solvents was associated with the “chronic productive cough” subclass (aMOR = 2.81, 1.26–6.24). No association was found for the “minimal cough” subclass (Tables 2 and 3).

The previously standard definitions of chronic bronchitis (CB) and chronic phlegm (CP) resembled the “chronic productive cough” subclass, and their occupational risks were also similar (Appendix S1).

### 3.3 Sensitivity Analyses

Correlations between occupational exposures are shown in Figure S2. Generally, there were moderate to high correlations between exposures in each group (i.e., dust/gases, pesticides, solvents), and between the dust/gases and other groups; but low correlations between pesticides and solvents/metals. Associations with consistent evidence from the main analysis (Tables 2, 3, and S8) and sensitivity analyses accounting for such correlations (Tables S10–S16) have been highlighted and summarised by cough outcomes in Table 4, and by occupational exposure in Table 5.

There was consistent evidence supporting associations between biological dust and “cough with allergies”; aromatic solvents and “chronic dry cough”; as well as herbicides, other solvents and “intermittent productive cough”, “chronic productive cough” (Tables S13–S15).

## 4 Discussion

This is the first population‐based study that has investigated associations between occupational exposures and a contemporary classification of cough subclasses. We found distinct occupational risks for different cough subclasses. Occupational exposure to biological dust was associated with “cough with allergies”, while aromatic solvents were associated with “chronic dry cough”. Such associations were not found when using standard cough definitions (i.e., chronic cough [CC], chronic bronchitis [CB], and chronic phlegm [CP]), as these definitions did not capture the “allergic” or “dry” features of cough. We also found consistent associations between other solvents (other than aromatic and chlorinated), herbicides and the two productive cough subclasses (“intermittent productive cough” and “chronic productive cough”), as well as the standard CB and CP outcomes.

We found strong and consistent associations between biological dust and the “cough with allergies” subclass. This is not surprising, as occupational exposure to biological dust is a well‐established risk factor for asthma and allergies [21], yet allergic cough has rarely been studied in that context. We did not find any association between biological dust (nor mineral dust or gases and fumes) and the standard cough definitions. The borderline association between biological dust and CP was attenuated when correlated agents (e.g., pesticides) were adjusted or excluded in the sensitivity analyses. These findings are consistent with the literature, as studies accounting for correlations between dust/fumes and other occupational exposures found attenuated associations between dust/fumes and cough outcomes [13, 14] while other studies had mixed findings [10, 12, 22, 23, 24], possibly due to their various subgroup analysis and uncontrolled confounding (including confounding introduced by correlated exposures) [9].

Occupations with high exposure to biological dust in our study population included construction and manufacturing labourers, building frame and related trades workers, and farmers. As shown in our study, almost all other agents correlated with biological dust, and such correlations have introduced both type I and type II errors to the results in the main analysis. In other words, when correlated agents were not accounted for, associations with biological dust were likely to be biased. The association was also likely to be attenuated when allergic and non‐allergic cough were analysed together.

We consistently found aromatic solvents to be associated with the “chronic dry cough” subclass, but no such associations with standard cough definitions. To our knowledge, no previous study has investigated dry cough, that is, without phlegm in general populations that could be directly compared with our results. Our study has uncovered an important finding as dry cough is commonly seen in undiagnosed/refractory chronic cough and cough hypersensitivities [25]. Occupations with high exposure to aromatic solvents in our study population included painting and construction labourers. Further exploration of the underlying mechanisms between aromatic solvents and a “chronic dry cough” subclass is warranted to develop specific prevention/treatment strategies for dry cough.

Conversely, exposures to solvents other than aromatic and chlorinated solvents were consistently associated with the two productive cough subclasses (“chronic productive” and “intermittent productive”). The dose–response effects were shown in both ways: significant associations were shown for cumulative exposures; and the association was stronger if the productive cough was chronic as opposed to intermittent. These findings were consistent with a previous study, which found a higher age‐ and smoking‐adjusted prevalence of chronic productive cough for people exposed to solvents [26]. By definition, the “chronic productive cough” subclass resembled the standard definition of CB, and the definition of CP is part of CB. As expected, we found similar associations between other solvents and CB, CP.

We also found an interaction by sex, as the dose–response relationship between cumulative exposure of other solvents and CB was only seen among males, but not females. These findings were somewhat consistent with the ECRHS (European Community Respiratory Health Survey) [13] which found associations between ever‐exposure to other solvents and incident CP among males, but not females. However, sex‐stratified analysis was not conducted for CB, and there was no overall association between solvents and CB in the ECRHS. Another population‐based study (Lifelines) did not find any association between solvents and CC, CB or CP, possibly due to the use of a composite solvent variable as opposed to individual solvent types and the very low prevalence of solvent exposure [14]. As shown in our study, individual solvent types were highly correlated with each other, confounding their associations with cough subclasses. Additionally, neither the ECRHS nor the Lifelines used cumulative exposure [13, 14] therefore, they were unlikely to reveal the dose–response association found in the current study.

In the present analysis, the productive cough subclasses and CB, CP were also associated with occupational exposure to pesticides, especially herbicides. We found consistent evidence for associations between herbicides and the “intermittent productive cough”, as well as CP. However, evidence was less consistent for “chronic productive cough”, CB, or other pesticides (i.e., insecticides, fungicides) with wider confidence intervals. Such inconsistencies and the wide 95% CIs may partially be explained by the low prevalence of CB (*n* = 96, 3%) in the current sample. The ECRHS also found no association between pesticides and CB, and the incidence of CB was even lower (1.7%) [13]. Conversely, an earlier follow‐up of a TAHS subgroup at age 45 years found occupational exposures to all pesticides (and herbicides, insecticides individually) were associated with CB and CP, and their prevalence of CB was 8.7% (246 of 1332) at age 45 years [27].

The Lifeline study found associations between ever‐high exposure to pesticides and incident CB, as well as incident CP [14]. This may be explained by the higher power caused by the less stringent definition of CB (productive cough for > 3 months per year, as opposed to > 3 months and > 2 years) [14]. Both the current study and the ECRHS study found interactions between sex, a pesticide and CP; however, the current analysis found herbicides (cumulative) to be associated with CP in males, while the ECRHS study found insecticides as well as fungicides (ever‐exposure, combined levels) to be associated with CP in females [13] This could be explained by the different exposure levels of pesticides and correlations between individual pesticides in the two studies, as TAHS participants were almost 10‐fold more likely to be ever‐exposed to a pesticide with stronger correlations within different types of pesticides, compared to the more urbanised participants in ECRHS [13].

Our findings of associations between other solvents, pesticides and the productive cough outcomes (i.e., “intermittent productive cough”, “chronic productive cough”, CB, and CP) are important as the two productive cough subclasses were associated with disadvantaged lifetime lung function trajectories [6]; and CB are known to be linked to premature death [4]. As 99% of participants exposed to pesticides or other solvents were co‐exposed to dust/gases, and exposures to dust/gases alone were not associated with any of these productive cough outcomes (Table S16), this observation could be due to either a main effect by other solvents/pesticides or interaction with gases/dust. Further studies are needed to fully understand the underlying pathophysiological mechanisms. In our study population, personal service workers (i.e., hairdressers, beauty therapists) and shop salespeople were specifically exposed to solvents other than aromatic and chlorinated types; and people exposed to pesticides were mostly farmers. A detailed job history for these occupations is important when assessing patients whose cough is productive.

Similar to the Lifelines study [14], we did not find associations between metal exposures and standard cough definitions. However, the ECRHS found ever‐exposure to metals to be associated with each of CC, CB, and CP.

### 4.1 Strengths and Limitations

One of the major strengths of the current study is the use of cough subclasses identified by latent class analysis for a general population. They captured “allergic” and “non‐productive or dry” cough which do not feature in the commonly used standard definitions of CC, CB, and CP [6]. We applied a JEM to measure occupational exposure, based on complete job histories, to minimise recall bias or differential information bias introduced by self‐reported exposures. We also used cumulative exposure to assess dose–response relationships. By using data from a prospective cohort study, we were able to control for multiple important confounders, including asthma status in childhood. We also used multiple sensitivity analyses to assess the effect of co‐exposures in our findings.

Despite the above strengths, there are some limitations. Although the use of JEM is well‐established in epidemiological studies, a major limitation is that it cannot account for individual variation in exposure levels for people who have the same job title. The group‐based exposure assessment results in Berkson‐type error that will result in no or little bias in risk estimates but with increased imprecision (i.e., wider 95% CIs) [28]. Both the cough outcomes and occupational exposures were collected at age 53 years, and we were unable to exclude participants who had cough that preceded their exposures. Although we adjusted for multiple confounders that were identified by a DAG and guided by literature review, there could always be residual/unknown confounding. While multiple comparisons increased the possibility of chance findings, most associations were consistent across different exposure measures (i.e., ever‐exposure, cumulative exposure) and sensitivity analyses. However, given the number of comparisons and small subgroup sizes, spurious associations cannot be ruled out. Ideally, our findings are replicated in other suitable cohort studies before causal inferences are made.

We did not further stratify the analysis of cough subclasses by sex or smoking, as there were already six cough subclasses, but we conducted interaction analysis using standard cough definitions to compare with other studies. In addition, TAHS is a homogeneous predominantly Caucasian sample born in 1961 in the state of Tasmania, Australia, which may limit the generalisability of our findings to other ethnically more diverse populations.

In conclusion, using novel cough subclasses in a middle‐aged population, subclass‐specific occupational risks were identified, suggesting different management and prevention strategies for different cough subclasses. We found associations between biological dust and allergic cough; aromatic solvents and chronic dry cough; herbicides and other solvents and productive cough. Such associations could not be fully identified just using standard cough definitions, that is, chronic cough, chronic bronchitis, and chronic phlegm. These findings strengthen the evidence base on occupationally acquired cough to inform the clinical assessment of patients and health protection strategies in the workplace.

## Author Contributions

**Jingwen Zhang:** formal analysis (lead), methodology (lead), writing – original draft (lead). **Jennifer L. Perret:** conceptualization (equal), supervision (equal), writing – review and editing (equal). **Dinh S. Bui:** formal analysis (supporting), methodology (supporting), supervision (supporting), writing – review and editing (equal). **Sheikh M. Alif:** formal analysis (supporting), writing – review and editing (equal). **Michael J. Abramson:** conceptualization (equal), funding acquisition (equal), supervision (supporting), writing – review and editing (equal). **Anne B. Chang:** conceptualization (equal), supervision (equal), writing – review and editing (equal). **Hans Kromhout:** formal analysis (supporting), methodology (supporting), writing – review and editing (equal). **Garun S. Hamilton:** funding acquisition (equal), writing – review and editing (equal). **Paul S. Thomas:** funding acquisition (equal), writing – review and editing (equal). **Bircan Erbas:** formal analysis (supporting), funding acquisition (equal), writing – review and editing (equal). **Bruce R. Thompson:** funding acquisition (equal), writing – review and editing (equal). **Melanie C. Matheson:** funding acquisition (equal), methodology (equal), writing – review and editing (equal). **E. Haydn Walters:** conceptualization (equal), funding acquisition (equal), investigation (equal), writing – review and editing (equal). **Caroline J. Lodge:** conceptualization (equal), funding acquisition (equal), investigation (equal), supervision (equal), writing – original draft (supporting), writing – review and editing (equal). **Shyamali C. Dharmage:** conceptualization (equal), funding acquisition (lead), investigation (equal), project administration (equal), supervision (lead), writing – original draft (supporting), writing – review and editing (equal).

## Ethics Statement

The study was approved by Human Ethics Review Committees at all participating institutions and written informed consent was obtained from all participants.

## Conflicts of Interest

J.L.P. received funds from GlaxoSmithKline (GSK) and AstraZeneca for unrelated research via institution. A.B.C. received grants from NHMRC (ID 1157228), Australian Medical Research Future Fund (ID 1169868) for unrelated research, and payment for BMJ Evidence for asthma, Up‐to‐Date for chapters on cough and bronchiectasis, and is a member of a DSMB for unlicensed vaccines, NHMRC Health Impact Committee, NHMRC Women in Science, ERS Guideline Committee. M.J.A. received grants from Sanofi, GSK, Pfizer, and Boehringer‐Ingelheim via institution and holds an honorary position at Woolcock Institute. S.M.A. received consulting fees from Australian Healthcare Associates, support from NHMRC for meeting attendance, and is deputy convenor of the Thoracic Society of Australia and New Zealand. S.C.D. received support from GSK and AstraZeneca for travelling and presentations at conferences. B.E. is an Editorial Board member of Respirology and a co‐author of this article. She was excluded from all editorial decision‐making related to the acceptance of this article for publication. All other authors (J.Z., D.S.B., H.K., G.S.H., P.S.T., B.E., B.R.T., M.C.M., E.H.W., and C.J.L.) have no conflicts of interest to declare.

## Supporting information


**Data S1.** Supporting Information.

**Visual Abstract** Occupational risks over 10 years for different cough subclasses among middle‐aged Australians

## Data Availability

Individual participant data can be provided on request to the corresponding author to anyone with a suitable proposal. Raw data are not widely available, but expressions of interest can be discussed with the corresponding author. The corresponding author on an individual basis. Data request instructions are available at https://tahs.com.au/data‐request/. Data for all participants in the Tasmanian Longitudinal Health Study (TAHS) are available from https://tahs.com.au/data‐request/. The proposal will be considered by the TAHS steering committee.
