# Supplementary material for: Associations Between Occupational Exposures and Cough Subclasses Among Middle‐Aged Australians
Source: Respirology. 2025 Apr 2;30(9):840–50. doi: 10.1111/resp.70040 (PMC12438006; doi:10.1111/resp.70040)
Supplement: Supplementary file 1 — Data S1. Supporting Information. [file RESP-30-840-s001.docx]

# Supplementary Materials

**Figure S1. Flow chart of participants included in the study.**


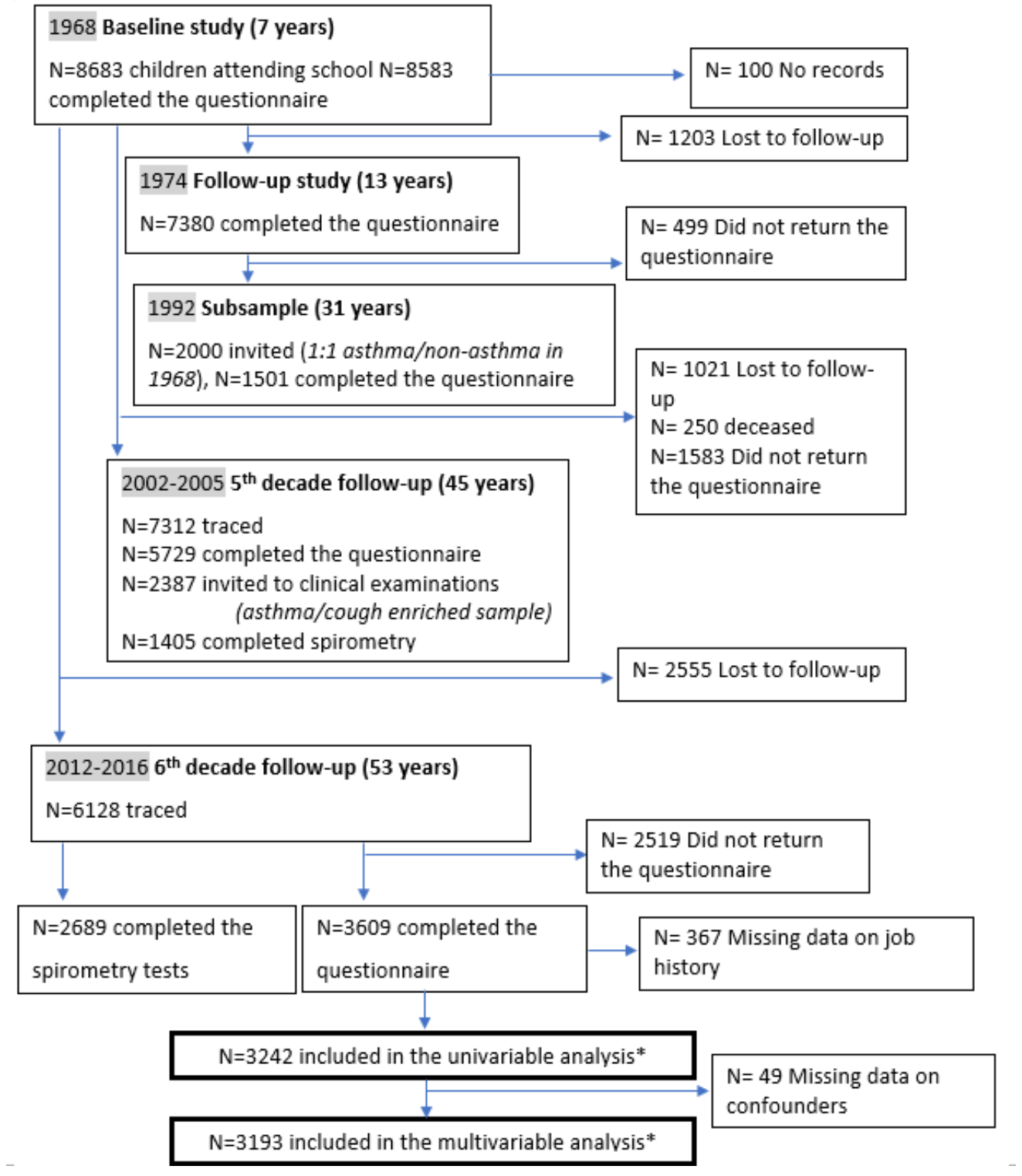


LCA: Latent class analysis. TAHS: Tasmanian Longitudinal Health Study. *There were 31 participants had incomplete data on duration of jobs, therefore excluded from the analysis using cumulative exposure unit years (N=3201 for univariable analysis, N=3152 for multivariable analysis).

**Figure S2. Correlation map (Spearman’s ρ) between occupational exposures in the study population**.

Colour-coding (-1 to 0 not shown):

**Appendix-1 Occupational exposure and previously standard cough definitions.**

At age 53 years, 10% (n=337) of the participants reported chronic cough (CC), 6% (n=189) chronic phlegm (CP), and 3% (n=96) chronic bronchitis (CB) (Table 1). Ever-high exposure to other solvents was associated with CB (adjusted odds ratio [aOR]=2.48, 95%CI: 1.01-6.06) and CP (aOR=2.26, 95%CI: 1.14-4.51). Cumulative exposure to herbicides was associated with CP (aOR=1.11, 95%CI: 1.03-1.20) (Table S6).

No evidence of interactions between the effects of occupational exposure and smoking, or between ever-exposures and sex on the standard cough definitions was found (data not shown). However, sex interacted with the effect of cumulative exposure to herbicides on CP, which was found especially in males (p-for-interaction=0.03; male aMOR=1.15, 95%CI: 1.06-1.24; female aMOR=0.86, 95%CI: 0.64-1.17). Sex also interacted with cumulative exposure to other solvents on CB, especially for females (p-for-interaction=0.10, males aMOR=0.94, 95%CI: 0.78-1.13; females: aMOR=1.29; 95%CI: 1.01-1.65) (Table S7).

**Table S1. Definitions of cough subclasses using latent class analysis (LCA).**

| **Cough Subclasses** | | | **Never coughers** | **Minimal cough** | **Cough with colds only** | **Cough with allergies** | **Intermittent productive cough** | | **Chronic dry cough** | **Chronic productive cough** |
| --- | --- | --- | --- | --- | --- | --- | --- | --- | --- | --- |
| N (%), total=3609 | | | 1396 (38.7%) | 206 (5.7%) | 1189 (32.9%) | 305 (8.5%) | 213 (5.9%) | | 147 (4.1%) | 153 (4.2%) |
| **Symptoms used in LCA** | | | Not included in LCA | **Conditional probabilities* of answering “Yes”** | | | | | | |
| Without colds | 1A. Usually cough <3 months/ year | |  | 0.85 | 0.14 | 0.20 | 0.56 | | 0.10 | 0.00 |
|  | 1B. Usually cough ≥3 months/ year | |  | 0.15 | 0.02 | 0.06 | 0.00 | | 0.90 | 1.00 |
|  | 2. Usually cough without colds > 5 years | |  | 0.07 | 0.00 | 0.00 | 0.07 | | 0.93 | 0.70 |
|  | 3A. Usually have phlegm <3 months/ year | |  | 0.10 | 0.05 | 0.05 | 0.99 | | 0.00 | 0.00 |
|  | 3B. Usually have phlegm ≥3 months/ year | |  | 0.00 | 0.00 | 0.00 | 0.01 | | 0.00 | 1.00 |
|  | 4.Usually have phlegm > 5 years | |  | 0.00 | 0.00 | 0.00 | 0.38 | | 0.00 | 0.63 |
| Related to colds | 5. Had cough with phlegm in the last year | |  | 0.03 | 0.25 | 0.17 | 0.29 | | 0.17 | 0.50 |
|  | 6. Colds usually goes to chest | |  | 0.00 | 0.71 | 0.41 | 0.67 | | 0.56 | 0.77 |
|  | 7. Chest illness producing phlegm <3 years | |  | 0.00 | 0.45 | 0.30 | 0.33 | | 0.29 | 0.52 |
| Allergic symptoms | 8. Start to cough when nearing animals | |  | 0.04 | 0.06 | 0.56 | 0.16 | | 0.32 | 0.23 |
|  | 9. Start to cough when nearing plants | |  | 0.00 | 0.01 | 0.55 | 0.13 | | 0.30 | 0.22 |
| **Cough/phlegm Symptoms** | | **Original survey questions used to define the symptoms** | | | | | | **Definition of “Yes”** | | |
| 1A. Usually cough <3 months/ year | | Q1. Do you usually cough when you do not have a cold? (Yes🡪 Q1a/No)  Q1a. Are there months in which you cough on most days? (Yes🡪 Q1b and Q1c/No)  Q1b. Do you cough on most days for at least three months of each year? (Yes/No)  Q1c. For how many years have you had this cough? (<2 years, 2-5 years, >5 years) | | | | | | “Yes” to Q1 and Q1a but “No” to Q1b. | | |
| 1B. Usually cough ≥3 months/ year | |  |  |  |  |  |  | “Yes” to Q1 and Q1a and Q1b. | | |
| 2. Usually cough without colds > 5 years | |  |  |  |  |  |  | “Yes” to Q1 and Q1a and “>5years” to Q1c | | |
| 3A. Usually have phlegm <3 months/ year | | Q2. Do you usually have phlegm in your chest when you do not have a cold? (Yes🡪 Q2a/No)  Q2a. Are there months in which you have phlegm in your chest on most days? (Yes🡪Q2b and Q2c/No)  Q2b. Do you bring up this phlegm on most days for at least three months of each year? (Yes/No)  Q2c. For how many years have you had this phlegm? (<2 years, 2-5 years, >5 years) | | | | | | “Yes” to Q2 and Q2a but “No” to Q2b. | | |
| 3B. Usually have phlegm ≥3 months/ year | |  |  |  |  |  |  | “Yes” to Q2 and Q2a and Q2b. | | |
| 4.Usually have phlegm > 5 years | |  |  |  |  |  |  | “Yes” to Q2 and Q2a and “>5years” to Q2c | | |
| 5. Had cough with phlegm in the last year | | Q3. Have you at any time in your life suffered from attacks of bronchitis or attacks of cough with phlegm (sputum) in the chest (‘loose’ or ‘rattly” cough)? (Yes🡪 Q3a/No)  Q3a. How long is it since the last attack? (within the last 6 months/within the last year but long than 6months/less or equal to 2 years but more than 1 years, more than 2 years) | | | | | | “Yes” to Q3  AND  “within the last 6months”/ “within the last year but long than 6months” to Q3a | | |
| 6. Colds usually goes to chest | | Q4. If you get a cold, does it usually go to your chest (Usually means more than half of the time)? (No/Yes/Do not get colds) | | | | | | “Yes” to Q4 (“Does not get colds” was considered as “No”) | | |
| 7. Chest illness producing phlegm <3 years | | Q5. During the past 3 years, have you had any chest illness that have kept you off work, indoors at home, or in bed? (Yes/No)  Q5a: Did you produce phlegm with any of the chest illness? (Yes/No) | | | | | | “Yes” to both Q5 and Q5a. | | |
| 8. Start to cough when nearing animals | | Q6. When you are near animals, such as cats, dogs or horses; near feathers, including pillows, quilts or doonas, or in a dusty part of house, do you ever start to cough? (Yes/No) | | | | | | “Yes” to Q6 | | |
| 9. Start to cough when nearing plants | | Q7. When you are near trees, grass or flowers, or when there is a lot of pollen about, do you ever start to cough? (Yes/No) | | | | | | “Yes” to Q7 | | |

* Latent class analysis was performed based on 9 cough-related questions among coughers (answered “Yes” to at least 1 of the 9 cough-related questions, definitions in Table S1, see supplements pp2). Those answered “Yes” to questions 1 and 3 were further stratified into “Yes but <3 months/ year” and “Yes and ≥3 months/ year”; and are shown in 1A/3A and 1B/3B, respectively.

Colour code of the heatmap showing conditional probabilities:

**Table S2. Cross-sectional and longitudinal features of the cough subclasses.**

| Cough subclasses | Never coughers | Minimal cough | Cough with colds only | Cough with allergies | Intermittent productive cough | | | Chronic dry cough | Chronic productive cough | |
| --- | --- | --- | --- | --- | --- | --- | --- | --- | --- | --- |
| Cross-sectional features (53 years) | No cough, reference group | Cough without colds, higher smoking rate | Recent respiratory infection and pneumonia | More females, highest allergic symptoms (CRS, asthma, hay fever, food allergy, and eczema), atopy*, lowest smoking rate | Highest BMI, higher smoking rate | | | Smoking rate comparable to the reference | Highest smoking rate | |
|  |  |  |  |  | Higher asthma, COPD, GORD, CRS, OSA, anxiety and depression. | | | | | |
| Longitudinal clinical features (**from age first noted**) that differed significantly from the reference group(s). | | | | | | | | | | |
| 7 years | Reference groups | | -- | Asthma**↑** | | FEV_1_ & FEV_1_/FVC trajectories | -- | | | Asthma**↑**, FEV_1_ & FEV_1_/FVC trajectories |
| 13 years |  |  | -- |  | | Asthma**↑** | -- | | | Productive cough**↑** |
| 31 years |  |  | -- |  | | Productive cough**↑** | -- | | |  |
| 43 years |  |  | Asthma**↑** | Productive cough**↑** | |  | Productive cough**↑**, Asthma**↑** | | | Smoking**↑**  FEV_1_/FVC**↓**,  FEV_1_**↓**, FVC **↓** |
| 53 years |  |  | Productive cough**↑**, FEV_1_/FVC**↓** | Smoking**↓**  FEV_1_**↓**  FEV_1_/FVC**↓** | | SHS**↑**, FEV_1_**↓**, FEV_1_/FVC, FVC **↓** | FEV_1_**↓**  FEV_1_/FVC**↓** | | | SHS**↑** |

*Atopy: defined by skin prick tests.

COPD: chronic obstructive pulmonary disease, GORD: gastro-oseophageal reflux disease, CRS: chronic rhinosinusitis, LCA: latent class analysis, OSA: obstructive sleep apnoea, SHS: second-hand smoke, FEV_1_: forced expiratory volume in the first one second, FVC: forced vital capacity. See detailed data and explanation in the published paper: Zhang, J., et al. (2024). Association of novel adult cough subclasses with clinical characteristics and lung function across six decades of life in a prospective, community-based cohort in Australia: An analysis of the Tasmanian Longitudinal Health Study (TAHS). *The Lancet Respiratory Medicine*, *12*(2), 129-140.

**Table S3. Definitions of potential confounders and characteristics.**

| Confounders | Variable type | Definitions/questions |
| --- | --- | --- |
| Chronic cough (CC) | Binary | “Yes” to both “Do you usually cough when you do not have a cold” AND “Do you cough on most days for at least three months of each year?” |
| Chronic phlegm (CP) | Binary | Yes” to both “Do you usually bring up phlegm when you do not have a cold” AND “Do you bring up this phlegm on most days for at least three months of each year? ” |
| Chronic bronchitis (CB) | Binary | CC AND CP AND answered “2-5years” or “more than 5 years” to both of the following questions “For how many years have you had this cough?”, “For how many years have you had this phlegm?” (less than 2 years/2-5 years/ more than 5 years). |
| Sex | Binary | Male/female |
| Education | Categorical | Three levels of education were defined based on the highest attained education reported, including lower to grade 12; completion of grade 12 or equivalent (trade or apprenticeship; certificate or diploma), and university or higher. |
| Smoking | Categorical | Current smokers, past smokers, and never smokers; based on answers to “Have you ever smoked cigarettes or any form of tobacco regularly?” and “Do you now smoke cigarettes?” |
| Pack-years | Continuous | The numbers of cigarettes smoked per day divided by 20 multiplied by the number of years of smoking |
| Asthma in childhood* | Binary | “Yes” to “Has she/he at any time in his/her life suffered from attacks of asthma or of wheezy breathing”? and had the last attack within 12 months |
| Asthma in adulthood | Binary | “Yes” to “Have you at any time suffered from attacks of asthma or of wheezy breathing”? and had the last attack within 12 months |

*Data collected at baseline study (mean age 7 years) and the 1974 follow-up study (mean age 13 years), having asthma in either study was considered having asthma in childhood. All other variables were defined using data collected from the 2012 follow-up study (mean age 53 years).

**Table S4. Features of participants with and without data for occupational exposure.**

P-values were calculated from Chi^2^ tests.

| Demographic characteristics | Participants with occupational data (N=3242) | Participants without occupational data (N=367) | P-values |
| --- | --- | --- | --- |
| Male | 1577 (48.6%) | 189 (51.5%) | 0.300 |
| Education |  |  | 0.067 |
| <Grade 12 | 1072 (33.4%) | 138 (37.7%) |  |
| Grade 12 or equivalent | 1376 (42.9%) | 134 (36.6%) |  |
| University or higher | 759 (23.7%) | 94 (25.7%) |  |
| Smoking |  |  | <0.001 |
| Never | 1432 (44.3%) | 132 (36.0%) |  |
| Past | 1246 (38.5%) | 143 (39.0%) |  |
| Current | 557 (17.2%) | 92 (25.1%) |  |
| Obesity (BMI>30 kg/m^2^) | 1046 (32.5%) | 104 (29.3%) | 0.215 |
| Adulthood asthma | 432 (13.4%) | 40 (10.9%) | 0.191 |
| Chronic cough | 337 (10.5%) | 30 (8.3%) | 0.190 |
| Chronic phlegm | 189 (5.9%) | 17 (4.7%) | 0.348 |
| Chronic bronchitis | 96 (3.0%) | 11 (3.0%) | 0.962 |
| Cough subclasses |  |  | 0.507 |
| Non coughers | 1242 (38.3%) | 154 (42.0%) |  |
| Minimal cough | 181 (5.6%) | 25 (6.8%) |  |
| Cough with colds only | 1069 (33.0%) | 120 (32.7%) |  |
| Cough with allergies | 281 (8.7%) | 24 (6.5%) |  |
| Intermittent productive cough | 195 (6.0%) | 18 (4.9%) |  |
| Chronic dry cough | 135 (4.2%) | 12 (3.3%) |  |
| Chronic productive cough | 139 (4.3%) | 14 (3.8%) |  |

**Table S5. Distributions of occupational exposures by age 53 years (N=3242).**

| Occupational exposures (N=3242) | | | | | |
| --- | --- | --- | --- | --- | --- |
| Groups | Agents | Ever-exposure, n (%) | | | Cumulative exposure unit-year, |
|  |  | Only-low | Ever-high | Total (only-low and ever-high) | median, IQR (n) |
| Dust/gases | Biological dust | 1178 (36%) | 629 (19%) | 1807 (56%) | 2, 3 (1749) |
|  | Mineral dust | 739 (23%) | 859 (27%) | 1598 (49%) | 3, 5 (1558) |
|  | Gases and fumes | 1431 (44%) | 890 (27%) | 2321 (72%) | 3, 4 (2270) |
|  | Dust/gases composite | 1162 (36%) | 1264 (39%) | 2426 (75%) | 3, 7 (2369) |
| Pesticides | Herbicides | 176 (5%) | 315 (10%) | 491 (15%) | 2, 4 (488) |
|  | Insecticides | 107 (3%) | 378 (12%) | 485 (15%) | 2, 3 (470) |
|  | Fungicides | 121 (4%) | 394 (12%) | 515 (16%) | 2, 4 (461) |
|  | All pesticides composite | 159 (5%) | 408 (13%) | 567 (17%) | 2, 4 (541) |
| Solvents | Aromatic | 768 (24%) | 61 (2%) | 829 (26%) | 1, 2 (806) |
|  | Chlorinated | 329 (10%) | 291 (9%) | 620 (19%) | 2, 4 (604) |
|  | Other solvents | 1172 (36%) | 109 (3%) | 1281 (40%) | 1, 2 (1243) |
| Metals | | 392 (12%) | 314 (10%) | 706 (22%) | 2, 5 (686) |

**Table S6. Distributions of ever occupational exposures stratified by cough subclasses (N=3242).**

| Cough outcomes | | | Cough subclasses | | | | | | | Standard cough definitions | | |
| --- | --- | --- | --- | --- | --- | --- | --- | --- | --- | --- | --- | --- |
| Ever-exposure n (%) | | | Non-coughers (n=1242) | Minimal cough (n=181) | Cough with colds only (n=1069) | Cough with allergies (n=281) | Intermittent productive cough (n=195) | Chronic dry cough (n=135) | Chronic productive cough (n=139) | CC (n=337) | CB (n=96) | CP (n=189) |
| Dust/gases | Biological dust | L | 418 (34%) | 66 (36%) | 399 (37%) | 126 (45%) | 75 (38%) | 40 (30%) | 54 (39%) | 126 (38%) | 36 (38%) | 74 (39%) |
|  |  | H | 242 (19%) | 32 (18%) | 190 (18%) | 62 (22%) | 38 (19%) | 34 (25%) | 31 (22%) | 74 (22%) | 25 (26%) | 43 (23%) |
|  | Mineral dust | L | 256 (21%) | 46 (25%) | 245 (23%) | 60 (21%) | 57 (29%) | 35 (26%) | 40(29%) | 92 (27%) | 33 (34%) | 56 (30%) |
|  |  | H | 316 (25%) | 47 (26%) | 295 (28%) | 76 (27%) | 52 (27%) | 34 (25%) | 39 (28%) | 87 (26%) | 26 (27%) | 53 (28%) |
|  | Gases and fumes | L | 523 (42%) | 80 (44%) | 470 (44%) | 139 (49%) | 97 (49%) | 57 (42%) | 65 (47%) | 155 (46%) | 45 (47%) | 92 (49%) |
|  |  | H | 319 (26%) | 54 (30%) | 305 (29%) | 74 (26%) | 58 (30%) | 37 (27%) | 43 (31%) | 94 (28%) | 32 (33%) | 61 (32%) |
| Pesticides | Herbicides | L | 67 (5%) | 6(3%) | 59 (6%) | 12 (6%) | 12 (6%) | 6 (4%) | 10 (7%) | 22 (7%) | 7 (7%) | 11 (6%) |
|  |  | H | 111 (9%) | 17 (9%) | 101 (10%) | 25 (13%) | 25 (13%) | 12 (9%) | 15 (11%) | 31 (9%) | 11 (11%) | 24 (13%) |
|  | Insecticides | L | 47 (4%) | 5 (3%) | 27 (3%) | 11 (4%) | 6 (3%) | 5 (4%) | 6 (4%) | 17 (5%) | 4 (4%) | 7 (4%) |
|  |  | H | 134 (11%) | 17 (9%) | 130 (12%) | 40 (14%) | 30 (15%) | 12 (9%) | 15 (11%) | 32 (10%) | 10 (10%) | 24 (13%) |
|  | Fungicides | L | 52 (4%) | 8 (4%) | 31 (3%) | 10 (4%) | 7 (4%) | 9 (7%) | 4 (3%) | 19 (6%) | 2 (2%) | 5 (3%) |
|  |  | H | 142 (11%) | 17 (9%) | 133 (12%) | 41 (15%) | 30 (15%) | 13 (10%) | 18 (13%) | 36 (11%) | 13 (14%) | 27 (14%) |
| Solvents | Aromatic solvents | L | 249 (22%) | 40 (22%) | 274 (26%) | 64 (23%) | 48 (25%) | 34 (25%) | 29 (21%) | 73 (22%) | 21 (22%) | 40 (21%) |
|  |  | H | 23 (2%) | 3 (2%) | 16 (2%) | 3 (1%) | 3 (2%) | 5 (4%) | 8 (6%) | 12 (4%) | 5 (5%) | 8 (4%) |
|  | Chlorinated solvents | L | 121 (10%) | 18 (10%) | 116 (11%) | 22 (8%) | 16 (8%) | 17 (13%) | 19 (14%) | 41 (12%) | 14 (15%) | 26 (14%) |
|  |  | H | 102 (8%) | 22 (12%) | 111 (10%) | 17 (6%) | 18 (9%) | 11 (8%) | 10 (7%) | 25 (7%) | 8 (8%) | 16 (9%) |
|  | Other solvents | L | 424 (34%) | 69 (38%) | 409 (38%) | 106 (38%) | 72 (37%) | 46 (34%) | 46 (33%) | 113 (34%) | 35 (36%) | 69 (37%) |
|  |  | H | 36 (3%) | 7 (4%) | 34 (3%) | 5 (2%) | 6 (3%) | 8 (6%) | 13 (9%) | 22 (7%) | 9 (9%) | 15 (8%) |
| Metals | | L | 157 (13%) | 20 (11%) | 130 (12%) | 22 (8%) | 21 (11%) | 23 (17%) | 19 (14%) | 49 (15%) | 13 (14%) | 24 (13%) |
|  |  | H | 107 (95) | 23 (13%) | 123 (12%) | 20 (7%) | 18 (9%) | 11 (8%) | 12 (9%) | 25 (7%) | 10 (10%) | 18 (10%) |

L: only-low exposure, H: ever-high exposure, as opposed to no exposure.

**Table S7. Distributions of cumulative occupational exposures stratified by cough subclasses.**

| Cough outcomes | | Cough subclasses | | | | | | | Standard cough definitions | | |
| --- | --- | --- | --- | --- | --- | --- | --- | --- | --- | --- | --- |
| Cumulative exposure-unit year | | Non-coughers | Minimal cough | Cough with colds only | Cough with allergies | Intermittent productive cough | Chronic dry cough | Chronic productive cough | CC | CB | CP |
| Dust/gases | Biological dust | 2.1 (3.0) | 2.0 (2.7) | 1.9 (2.7) | 1.95 (2.90) | 2.1 (3.1) | 2.5 (3.0) | 1.8 (2.9) | 1.8 (2.9) | 1.8 (2.9) | 1.9 (2.7) |
|  | Mineral dust | 2.9 (5.3) | 2.8 (3.7) | 2.6 (5.5) | 1.85 (3.60) | 2.6 (4.5) | 1.9 (3.6) | 2.2 (4.3) | 2.1 (3.6) | 2.1 (4.6) | 2.1 (4.7) |
|  | Gases and fumes | 2.7 (3.8) | 3.0 (3.3) | 2.7 (3.5) | 2.10 (3.10) | 2.8 (3.7) | 2.8 (3.3) | 2.5 (3.1) | 2.6 (3.2) | 2.7 (3.5) | 2.7 (3.4) |
| Pesticides | Herbicides | 2.0 (3.2) | 2.5 (4.2) | 1.3 (3.0) | 1.78 (3.00) | 2.0 (5.1) | 1.6 (4.0) | 1.2 (2.7) | 1.2 (3.3) | 1.2 (4.7) | 2.1 (5.6) |
|  | Insecticides | 2.3 (3.8) | 1.8 (3.8) | 1.8 (3.0) | 1.60 (4.40) | 2.2 (4.8) | 1.8 (4.0) | 1.5 (3.5) | 1.7 (3.6) | 1.8 (5.1) | 3.2 (5.0) |
|  | Fungicides | 2.2 (3.9) | 1.5 (3.2) | 1.8 (3.0) | 1.70 (4.20) | 2.5 (4.3) | 1.7 (3.7) | 2.2 (4.6) | 1.9 (4.2) | 3.6 (5.9) | 3.2 (5.6) |
| Solvents | Aromatic solvents | 1.2 (2.1) | 1.4 (2.8) | 1.3 (2.0) | 1.2 (2.4) | 1.4 (2.1) | 1.2 (2.4) | 1.2 (1.9) | 1.2 (2.7) | 1.0 (1.8) | 1.2 (2.1) |
|  | Chlorinated solvents | 2.0 (5.0) | 2.2 (5.5) | 2.1 (5.1) | 1.4 (4.1) | 3.1 (4.0) | 2.3 (2.8) | 1.2 (2.6) | 2.0 (2.9) | 1.0 (2.5) | 1.0 (3.0) |
|  | Other solvents | 1.4 (2.5) | 1.5 (2.7) | 1.5 (2.2) | 1.5 (2.2) | 1.7 (2.3) | 1.8 (3.1) | 1.2 (2.2) | 1.3 (2.6) | 1.2 (1.7) | 1.4 (2.2) |
| Metals | | 1.6 (3.8) | 3.0 (8.6) | 2.1 (5.4) | 1.6 (4.7) | 2.5 (4.7) | 1.6 (3.3) | 1.5 (2.2) | 1.6 (3.2) | 1.5 (2.2) | 1.6 (2.1) |

IQR: inter quartile range. Numbers presented as median, IQR. Medians and IQRs were calculated only among participants with values >0.

**Table S8. Adjusted associations between occupational exposures (ever-exposure) and standard cough definitions (chronic cough, chronic bronchitis, chronic phlegm) at age 53 years.**

| Standard cough definitions at 53 years | | Ever-exposure, aOR (95%CI), p-value | | | | | | Cumulative exposure unit-year, aOR for per 10 exposure unit-year increase (95% CI), p-value | | | | |
| --- | --- | --- | --- | --- | --- | --- | --- | --- | --- | --- | --- | --- |
|  |  | CC (n=3004) | | CB (n=3005) | | CP (n=2998) | | CC (n=2966) | | CB (n=2967) | | CP (n=2960) |
| Biological dust | L | | 0.93 (0.70-1.23) | | 0.85 (0.50-1.44) | | 1.00 (0.69-1.45) | | 0.99 (0.95-1.03) | 1.03 (0.96-1.10) | | 1.03 (0.98-1.08) |
|  | H | | 1.06 (0.75-1.48) | | 1.23 (0.70-2.19) | | 1.04 (0.67-1.62) | |  |  |  |  |
| Mineral dust | L | | 1.12 (0.82-1.53) | | 1.53 (0.90-2.62) | | 1.15 (0.7-1.73) | | 0.99 (0.95-1.03) | 1.00 (0.93-1.06) | | 1.00 (0.95-1.05) |
|  | H | | 0.90 (0.64-1.27) | | 0.89 (0.48-1.67) | | 0.89 (0.57-1.39) | |  |  |  |  |
| Gases and fumes | L | | 0.97 (0.72-1.31) | | 1.24 (0.69-2.23) | | 1.38 (0.90-2.12) | | 1.01 (0.97-1.05) | 1.01 (0.95-1.07) | | 1.02 (0.97-1.06) |
|  | H | | 0.87 (0.60-1.25) | | 1.12 (0.57-2.19) | | 1.26 (0.76-2.07) | |  |  |  |  |
| Herbicides | L | | 1.18 (0.71-1.97) | | 1.18 (0.50-2.79) | | 0.77 (0.37-1.62) | | 0.98 (0.90-1.07) | 1.04 (0.92-1.18) | **1.11 (1.03-1.20)** | |
|  | H | | 0.88 (0.58-1.35) | | 1.03 (0.51-2.09) | | 1.27 (0.77-2.07) | |  |  |  |  |
| Insecticides | L | | 1.60 (0.89-2.87) | | 1.24 (0.42-3.64) | | 0.96 (0.40-2.31) | | 0.95 (0.88-1.04) | 0.98 (0.86-1.11) | 1.03 (0.95-1.12) | |
|  | H | | 0.74 (0.49-1.12) | | 0.74 (0.36-1.50) | | 0.90 (0.55-1.48) | |  |  |  |  |
| Fungicides | L | | 1.60 (0.92-2.77) | | 0.48 (0.11-2.05) | | 0.51 (0.18-1.44) | | 0.99 (0.92-1.06) | 1.02 (0.92-1.13) | 1.05 (0.97-1.13) | |
|  | H | | 0.83 (0.56-1.24) | | 0.96 (0.50-1.82) | | 1.00 (0.62-1.60) | |  |  |  |  |
| Aromatic solvents | L | | 0.87 (0.63-1.21) | | 0.87 (0.49-1.55) | | 0.68 (0.44-1.05) | | 1.06 (0.97-1.16) | 0.93 (0.77-1.12) | 0.98 (0.86-1.11) | |
|  | H | | 1.84 (0.91-3.72) | | 2.10 (0.73-6.05) | | 1.68 (0.72-3.93) | |  |  |  |  |
| Chlorinated solvents | L | | 1.13 (0.76-1.69) | | 1.37 (0.70-2.68) | | 1.22 (0.74-2.04) | | 0.98 (0.92-1.04) | 0.96 (0.86-1.09) | 0.95 (0.86-1.04) | |
|  | H | | 0.78 (0.48-1.29) | | 1.04 (0.46-2.38) | | 0.88 (0.47-1.65) | |  |  |  |  |
| Other solvents | L | | 0.87 (0.67-1.14) | | 1.06 (0.66-1.70) | | 1.06 (0.75-1.50) | | 1.04 (0.97-1.11) | 1.03 (0.92-1.16) | 1.07 (0.98-1.15) | |
|  | H | | 1.60 (0.89-2.89) | | **2.48 (1.01-6.06)** | | **2.26 (1.14-4.51)** | |  |  |  |  |
| Metals | L | | 1.17 (0.79-1.72) | | 0.87 (0.43-1.77) | | 0.86 (0.50-1.46) | | 0.96 (0.91-1.02) | 0.97 (0.87-1.07) | 0.93 (0.85-1.02) | |
|  | H | | 0.68 (0.41-1.12) | | 0.92 (0.42-2.03) | | 0.74 (0.40-1.37) | |  |  |  |  |

CC: chronic cough, cough ≥ 3 months; CB: cough and phlegm ≥ 3 months and ≥ 2 years; CP: chronic phlegm, phlegm ≥ 3 months. aOR: adjusted relative risk, adjusted for sex, education, smoking, pack-years, childhood asthma and adulthood asthma at age 53 years. L: only-low; H: ever-high exposure; no exposure to any of the agents as the reference. Bolded results for P<0.05

**Table S9. Interaction between sex, cumulative exposure on standard cough definitions.**

| Exposure & outcome | Sex-stratified associations | | Sex-stratified associations after adjusting for correlated agents* |
| --- | --- | --- | --- |
|  | P-for-interaction | aOR, 95%CI, p-values | aOR, 95%CI, p-values |
| Biological dust  CP | 0.011 | M: 1.06 (1.01-1.11) 0.027  F: 0.86 (0.73-1.01) 0.069 | M: 1.02 (0.96-1.09) 0.458  F: 0.82 (0.65-1.03) 0.089 |
| Herbicides  CP | 0.026 | M: 1.15 (1.06-1.24) 0.001  F: 0.86 (0.64-1.17) 0.341 | M: 1.44 (1.16-1.78) 0.001  F: 0.89 (0.51-1.56) 0.680 |
| Other solvents  CB | 0.095 | M: 0.94 (0.78-1.13) 0.498  F: 1.16 (1.02-1.33) 0.028 | M: 1.03 (0.70-1.50) 0.890  F: 1.29 (1.01-1.65) 0.040 |

aOR: adjusted odds ratio, adjusted for sex, education, smoking, pack-years, childhood asthma and adulthood asthma at age 53 years; cumulative exposure unit-year: cumulative exposure-unit year, aOR shown as per 10-year increase in cumulative exposure unit-year; CB: chronic bronchitis, CP: chronic phlegm; F: female, M:male.

Results not shown for p-for interaction ≥0.1.

* Correlated agents were adjusted for each other if ρ ≥ 0.3, composite variables were used if relevant, adjusted agents were listed in brackets as follows: biological dust (mineral dust, gases and fumes, all pesticides, other solvents); herbicides (fungicides, insecticides, Dust/gases), other solvents (Dust/gases, aromatic solvents, chlorinated solvents, metals).

**Table S10. Adjusted associations between occupational exposures (ever-exposure) and cough subclasses at age 53 years, multi-variant model adjusted for correlated agents (ρ ≥ 0.3).**

| Ever occupational exposures | | | Cough subclasses at age 53 years (N=3022) | | | | | |
| --- | --- | --- | --- | --- | --- | --- | --- | --- |
|  |  |  | Minimal cough (n=173) | Cough with colds only (n=1001) | Cough with allergies (n=264) | Intermittent productive cough (n=178) | Chronic dry cough (n=122) | Chronic productive cough (n=129) |
|  |  |  | aMOR (95%CI), p-value | | | | | |
| Dust/gases group | Biological dust | L | 0.84 (0.53-1.32) 0.446 | 0.94 (0.73-1.20) 0.616 | 1.43 (0.94-2.17) 0.094 | 0.82 (0.52-1.31) 0.411 | 0.77 (0.44-1.36) 0.370 | 0.75 (0.43-1.32) 0.321 |
|  |  | H | 0.72 (0.37-1.40) 0.325 | 0.76 (0.53-1.10) 0.147 | 1.59 (0.87-2.90) 0.128 | 0.57 (0.29-1.12) 0.104 | 1.47 (0.69-3.11) 0.313 | 0.74 (0.33-1.67) 0.466 |
|  | Mineral dust | L | 1.16 (0.71-1.91) 0.554 | 1.10 (0.84-1.45) 0.485 | 1.02 (0.67-1.55) 0.936 | 1.25 (0.77-2.02) 0.364 | 1.31 (0.71-2.40) 0.389 | 1.13 (0.62-2.05) 0.692 |
|  |  | H | 1.07 (0.53-2.19) 0.843 | 1.13 (0.77-1.67) 0.537 | 1.37 (0.71-2.65) 0.348 | 0.66 (0.31-1.40) 0.280 | 0.95 (0.39-2.31) 0.919 | 0.93 (0.39-2.23) 0.868 |
|  | Gases and fumes | L | 1.025 (0.74-2.13) 0.399 | 1.09 (0.83-1.45) 0.534 | 1.33 (0.84-2.12) 0.222 | 1.69 (0.98-2.92) 0.058 | 0.94 (0.50-1.74) 0.835 | 1.38 (0.72-2.64) 0.337 |
|  |  | H | 1.58 (0.76-3.27) 0.222 | 1.10 (0.74-1.64) 0.644 | 1.54 (0.79-2.99) 0.202 | 1.71 (0.82-3.59) 0.155 | 0.90 (0.37-2.17) 0.808 | 1.27 (0.52-3.11) 0.595 |
| Pesticide group | Herbicides | L | 0.97 (0.29-3.26) 0.957 | 1.21 (0.62-2.35) 0.583 | 1.48 (0.54-4.05) 0.442 | 0.92 (0.25-3.33) 0.899 | 0.92 (0.25-3.33) 0.899 | 2.98 (0.91-9.73) 0.071 |
|  |  | H | 2.34 (0.54-10.15) 0.256 | 0.86 (0.38-1.98) 0.727 | 1.85 (0.51-6.63) 0.347 | 1.73 (0.55-5.45) 0.345 | 2.32 (0.45-12.01) 0.315 | 4.82 (1.15-20.14) 0.031 |
|  | Insecticides | L | 0.48 (0.09-2.42) 0.373 | 0.76 (0.28-2.05) 0.586 | 1.24 (0.29-5.26) 0.769 | 0.86 (0.15-4.88) 0.867 | 0.22 (0.04-1.11) 0.067 | 1.97 (0.39-10.10) 0.414 |
|  |  | H | 5.65 (0.03-938.51) 0.507 | 3.11 (0.66-14.75) 0.153 | 0.80 (0.10-6.69) 0.838 | 3.52 (0.10-130.18) 0.494 | 0.23 (0.02-2.79) 0.249 | 0.11 (0.02-0.80) 0.029 |
|  | Fungicides | L | 1.72 (0.87-2.03) 0.184 | 0.70 (0.29-1.75) 0.450 | 0.56 (0.13-2.50) 0.447 | 0.64 (0.12-3.30) 0.596 | 3.57 (1.03-12.43) 0.046 | 0.19 (0.03-1.24) 0.082 |
|  |  | H | 1.11 (0.66-1.88) 0.697 | 0.37 (0.09-1.55) 0.172 | 0.90 (0.12-6.44) 0.913 | 0.14 (0.00-4.92) 0.278 | 1.47 (0.17-12.52) 0.722 | 1.92 (0.33-11.07) 0.468 |
| Solvent group | Aromatic solvents | L | 0.78 (0.42-1.45) 0.429 | 1.18 (0.86-1.64) 0.309 | 1.50 (0.92-2.44) 0.103 | 1.13 (0.64-2.00) 0.680 | 1.49 (0.74-2.99) 0.263 | 1.04 (0.51-2.09) 0.919 |
|  |  | H | 0.79 (0.13-4.75) 0.797 | 0.61 (0.24-1.60) 0.319 | 0.66 (0.06-7.49) 0.737 | 0.51 (0.09-2.98) 0.451 | 1.55 (0.20-11.79) 0.670 | 0.94 (0.19-4.72) 0.936 |
|  | Chlorinated solvents | L | 1.16 (0.52-2.56) 0.722 | 1.00 (0.66-1.50) 0.984 | 0.67 (0.34-1.33) 0.254 | 0.63 (0.27-1.47) 0.290 | 0.82 (0.35-1.96) 0.663 | 0.77 (0.29-2.00) 0.586 |
|  |  | H | 1.53 (0.49-4.78) 0.464 | 0.93 (0.49-1.74) 0.815 | 0.59 (0.19-1.85) 0.369 | 1.34 (0.42-4.30) 0.618 | 0.75 (0.19-3.02) 0.684 | 1.07 (0.28-4.04) 0.922 |
|  | Other solvents | L | 1.09 (0.69-1.72) 0.714 | 1.07 (0.83-1.38) 0.586 | 1.00 (0.69-1.46) 0.993 | 1.04 (0.67-1.63) 0.855 | 0.90 (0.51-1.59) 0.709 | 1.04 (0.61-1.78) 0.887 |
|  |  | H | 0.86 (0.18-4.06) 0.847 | 1.44 (0.64-3.21) 0.378 | 0.46 (0.08-2.81) 0.401 | 2.10 (0.47-9.42) 0.332 | 1.42 (0.20-9.92) 0.721 | 3.65 (0.74-18.06) 0.113 |
| Metals | | L | 0.93 (0.49-1.76) 0.824 | 0.89 (0.64-1.25) 0.508 | 0.69 (0.38-1.25) 0.219 | 0.65 (0.35-1.22) 0.182 | 1.33 (0.67-2.63) 0.421 | 0.82 (0.40-1.69) 0.597 |
|  |  | H | 1.13 (0.37-3.47) 0.831 | 1.24 (0.67-2.28) 0.501 | 1.06 (0.36-3.15) 0.915 | 0.48 (0.15-1.54) 0.219 | 1.01 (0.26-3.88) 0.987 | 1.10 (0.57-2.10) 0.778 |

aMOR: adjusted multinomial odds ratio, adjusted for sex, education, smoking, pack-years, childhood asthma and adulthood asthma at age 53 years. L: only-low; H: ever-high; no exposure to any of the agents as the reference; all compared to the “non-coughers” as the reference group. Correlated agents were adjusted for each other if ρ ≥ 0.3, composite variables were used if relevant, ); pesticides were adjusted for each other and Dust/gases; solvents and metals were adjusted for each other and Dust/gases; the adjusted agents for the Dust/gases group were listed in brackets as follows: biological dust (mineral dust, gases and fumes, all pesticides, other solvents), mineral dust (biological dust, gases and fumes, all pesticides, metals, solvent group), gases and fumes (biological dust, mineral dust, all pesticides, metals, solvent group), Dust/gases composite (all pesticides, metals, solvent group).

**Table S11. Adjusted associations between occupational exposures (cumulative exposure unit-year) and cough subclasses at age 53 years, multi-variant model adjusted for correlated agents (ρ ≥ 0.3).**

| Cumulative occupational exposures | | Cough subclasses at age 53 years (N=2984) | | | | | |
| --- | --- | --- | --- | --- | --- | --- | --- |
|  |  | Minimal cough (n=170) | Cough with colds only (n=991) | Cough with allergies (n=263) | Intermittent productive cough (n=174) | Chronic dry cough (n=118) | Chronic productive cough (n=127) |
|  |  | aMOR for per 10 exposure unit-year increase (95% CI), p-value | | | | | |
| Dust/gases group | Biological dust | 0.99 (0.93-1.06) 0.831 | 0.99 (0.95-1.02) 0.486 | 1.05 (0.99-1.11) 0.078 | 0.97 (0.90-1.03) 0.335 | 0.98 (0.90-1.06) 0.625 | 0.97 (0.89-1.05) 0.440 |
|  | Mineral dust | 0.96 (0.90-1.03) 0.285 | 1.01 (0.97-1.04) 0.771 | 1.00 (0.93-1.07) 0.971 | 0.91 (0.4-0.98) 0.014 | 0.89 (0.80-0.99) 0.034 | 0.97 (0.89-1.06) 0.490 |
|  | Gases and fumes | 1.03 (0.97-1.11) 0.309 | 0.99 (0.96-1.03) 0.647 | 0.96 (0.89-1.03) 0.230 | 1.04 (0.97-1.11) 0.292 | 1.07 (0.98-1.16) 0.119 | 1.02 (0.94-1.10) 0.720 |
| Pesticide group | Herbicides | 1.14 (0.93-1.39) 0.204 | 0.97 (0.86-1.10) 0.654 | 1.00 (0.84-1.18) 0.999 | 1.23 (1.04-1.48) 0.017 | 1.14 (0.87-1.49) 0.334 | 1.21 (0.97-1.51) 0.094 |
|  | Insecticides | 1.20 (0.61-2.37) 0.594 | 1.16 (0.92-1.45) 0.211 | 1.90 (0.84-4.29) 0.122 | 1.19 (0.64-2.19) 0.584 | 0.71 (0.51-0.98) 0.037 | 0.75 (0.55-1.01) 0.057 |
|  | Fungicides | 0.73 (0.38-1.40) 0.339 | 0.87 (0.71-1.08) 0.207 | 0.58 (0.26-1.30) 0.184 | 0.75 (0.42-1.36) 0.349 | 1.17 (0.97-1.42) 0.098 | 1.14 (0.092-1.43) 0.227 |
| Solvent group | Aromatic solvents | 0.96 (0.79-1.15) 0.632 | 1.00 (0.90-1.11) 0.969 | 1.03 (0.87-1.23) 0.711 | 0.93 (0.78-1.10) 0.388 | 1.17 (0.96-1.42) 0.114 | 0.96 (0.80-1.16) 0.692 |
|  | Chlorinated solvents | 0.97 (0.85-1.11) 0.671 | 1.00 (0.92-1.08) 0.933 | 0.94 (0.81-1.08) 0.374 | 0.99 (0.84-1.16) 0.879 | 1.00 (0.81-1.22) 0.986 | 0.97 (0.80-1.17) 0.763 |
|  | Other solvents | 1.04 (0.93-1.17) 0.474 | 1.01 (0.94-1.08) 0.789 | 0.99 (0.89-1.11) 0.927 | 1.11 (1.00-1.24) 0.040 | 1.01 (0.86-1.18) 0.931 | 1.13 (1.00-1.28) 0.046 |
| Metals | | 1.08 (0.95-1.22) 0.260 | 1.04 (0.96-1.13) 0.315 | 1.05 (0.91-1.20) 0.509 | 0.99 (0.85-1.14) 0.863 | 0.96 (0.79-1.18) 0.723 | 0.98 (0.93-1.04) 0.862 |

aMOR: adjusted multinomial odds ratio, adjusted for sex, education, smoking, pack-years, childhood asthma and adulthood asthma at age 53 years. Cumulative exposure unit-year: cumulative exposure-unit year; all compared to the “non-coughers” as the reference group. Correlated agents were adjusted for each other if ρ ≥ 0.3, composite variables were used if relevant; pesticides were adjusted for each other and Dust/gases; solvents and metals were adjusted for each other and Dust/gases; the adjusted agents for the Dust/gases group were listed in brackets as follows: biological dust (mineral dust, gases and fumes, all pesticides, other solvents), mineral dust (biological dust, gases and fumes, all pesticides, metals, aromatic solvents), gases and fumes (biological dust, mineral dust, metals, solvent group), Dust/gases composite (all pesticides, solvent group).

**Table S12. Adjusted associations between occupational exposures and standard cough definitions at age 53 years, multi-variant model adjusted for correlated agents (ρ ≥ 0.3).**

| Standard cough definitions at 53 years | | | Ever-exposure, aMOR (95%CI), p-value | | | Cumulative exposure unit-year, aMOR for per 10 exposure unit-year increase (95% CI), p-value | | |
| --- | --- | --- | --- | --- | --- | --- | --- | --- |
|  |  |  | Chronic cough (n=3004) | Chronic bronchitis (n=3005) | Chronic phlegm (n=2998) | Chronic cough (n=2966) | Chronic bronchitis (n=2967) | Chronic phlegm (n=2960) |
| Dust/gases group | Biological dust | L | 0.98 (0.69-1.40) 0.914 | 0.73 (0.38-1.38) 0.333 | 0.86 (0.54-1.35) 0.505 | 0.98 (0.93-1.03) 0.440 | 1.03 (0.93-1.10) 0.753 | 0.99 (0.93-1.06) 0.833 |
|  |  | H | 1.17 (0.70-1.94) 0.548 | 1.08 (0.45-2.62) 0.860 | 0.90 (0.47-1.76) 0.787 |  |  |  |
|  | Mineral dust | L | 1.14 (0.78-1.66) 0.496 | 1.41 (0.72-2.76) 0.318 | 1.01 (0.63-1.64) 0.954 | 0.99 (0.94-1.05) 0.852 | 0.97 (0.88-1.06) 0.488 | 0.96 (0.89-1.03) 0.266 |
|  |  | H | 0.98 (0.56-1.71) 0.939 | 0.71 (0.26-1.97) 0.516 | 0.75 (0.36-1.56) 0.442 |  |  |  |
|  | Gases and fumes | L | 0.96 (0.64-1.43) 0.827 | 1.15 (0.53-2.50) 0.725 | 1.51 (0.87-2.62) 0.144 | 1.03 (0.97-1.08) 0.322 | 1.02 (0.93-1.12) 0.647 | 1.03 (0.97-1.11) 0.316 |
|  |  | H | 0.90 (0.51-1.58) 0.721 | 0.95 (0.34-2.61) 0.914 | 1.40 (0.67-2.94) 0.373 |  |  |  |
| Pesticide group | Herbicides | L | 1.42 (0.67-3.01) 0.362 | 2.50 (0.72-8.72) 0.151 | 1.77 (0.62-5.06) 0.286 | 1.09 (0.94-1.27) 0.273 | 1.16 (0.95-1.42) 0.13 | 1.33 (1.13-1.56) 0.001 |
|  |  | H | 2.22 (0.84-5.85) 0.108 | 4.39 (1.05-18.37) 0.043 | 5.94 (1.85-19.11) 0.003 |  |  |  |
|  | Insecticides | L | 1.00 (0.36-2.74) 0.998 | 2.84 (0.55-14.59) 0.211 | 2.34 (0.60-9.21) 0.223 | 0.76 (0.62-0.94) 0.011 | 0.71 (0.54-0.93) 0.014 | 0.74 (0.57-0.95) 0.019 |
|  |  | H | 0.14 (0.03-0.57) 0.006 | 0.05 (0.01-3.18) 0.002 | 0.08 (0.02-0.46) 0.004 |  |  |  |
|  | Fungicides | L | 1.35 (0.52-3.52) 0.544 | 0.12 (0.01-1.04) 0.054 | 0.21 (0.04-1.07) 0.061 | 1.17 (1.01-1.37) 0.042 | 1.24 (1.02-1.51) 0.034 | 1.13 (0.92-1.38) 0.236 |
|  |  | H | 2.75 (0.78-9.62) 0.114 | 4.15 (0.78-22.14) 0.096 | 2.62 (0.57-12.09) 0.218 |  |  |  |
| Solvent group | Aromatic solvents | L | 0.93 (0.60-1.46) 0.768 | 0.81 (0.37-1.76) 0.595 | 0.59 (0.32-1.07) 0.083 | 1.06 (0.94-1.20) 0.353 | 0.85 (0.68-1.05) 0.133 | 0.91 (0.78-1.05) 0.186 |
|  |  | H | 1.31 (0.39-4.33) 0.663 | 0.88 (0.14-5.37) 0.889 | 0.84 (0.21-3.23) 0.798 |  |  |  |
|  | Chlorinated solvents | L | 0.96 (0.53-1.74) 0.899 | 1.05 (0.36-3.03) 0.930 | 1.11 (0.49-2.49) 0.808 | 1.01 (0.89-1.14) 0.891 | 0.98 (0.80-1.19) 0.805 | 0.97 (0.82-1.14) 0.696 |
|  |  | H | 1.37 (0.56-3.35) 0.494 | 1.18 (0.29-4.85) 0.817 | 1.50 (0.50-4.53) 0.480 |  |  |  |
|  | Other solvents | L | 0.95 (0.67-1.34) 0.758 | 1.17 (0.64-2.12) 0.608 | 1.21 (0.79-1.87) 0.381 | 1.02 (0.93-1.12) 0.661 | 1.13 (0.99-1.28) 0.072 | 1.13 (1.03-1.25) 0.010 |
|  |  | H | 1.37 (0.44-4.27) 0.587 | 2.65 (0.44-16.04) 0.289 | 2.12 (0.57-7.89) 0.261 |  |  |  |
| Metals | | L | 1.17 (0.70-1.84) 0.507 | 0.77 (0.34-1.73) 0.524 | 0.84 (0.87-2.38) 0.153 | 0.94 (0.84-1.06) 0.305 | 0.99 (0.084-1.16) 0.909 | 0.95 (0.83-1.09) 0.485 |
|  |  | H | 0.56 (0.23-1.35) 0.198 | 0.84 (0.23-3.05) 0.789 | 0.66 (0.23-1.89) 0.443 |  |  |  |

aMOR: adjusted multinomial odds ratio, adjusted for sex, education, smoking, pack-years, childhood asthma and adulthood asthma at age 53 years. L: only-low; H: ever-high; no exposure to any of the agents as the reference.All compared to a common reference group with participants unexposed to any of the agent (i.e., no ever-exposure or cumulative exposure unit-year=0). Correlated agents were adjusted for each other if 1, pesticides were adjusted for each other and Dust/gases; solvents and metals were adjusted for each other and Dust/gases; the adjusted agents for the Dust/gases group were listed in brackets as follows: biological dust (mineral dust, gases and fumes, all pesticides, other solvents), mineral dust ever-exposure (biological dust, gases and fumes, all pesticides, metals, solvent group), mineral dust cumulative exposure unit-year (biological dust, gases and fumes, aromatic solvents, metals), gases and fumes ever-exposure (biological dust, mineral dust, all pesticides, metals, solvent group), gases and fumes cumulative exposure unit-year (biological dust, mineral dust, solvent group, metals), Dust/gases composite (all pesticides, metals, solvent group).

**Table S13. Adjusted associations between occupational exposures (ever-exposure) and cough subclasses at age 53 years, compared to fully unexposed group.**

| Ever occupational exposures | | | Cough subclasses at age 53 years | | | | | |
| --- | --- | --- | --- | --- | --- | --- | --- | --- |
|  |  |  | Minimal cough | Cough with colds only | Cough with allergies | Intermittent productive cough | Chronic dry cough | Chronic productive cough |
|  |  |  | aMOR (95%CI), p-value | | | | | |
| Dust/gases group | Biological dust  N=2438 | L | 1.19 (0.77-1.84) 0.434 | 1.14 (0.91-1.42) 0.257 | 1.67 (1.16-2.40) 0.006 | 1.40 (0.88-2.23) 0.158 | 0.73 (0.45-1.21) 0.226 | 1.04 (0.60-1.80) 0.884 |
|  |  | H | 1.01 (0.58-1.74) 0.978 | 0.93 (0.70-1.23) 0.606 | 1.79 (1.14-2.82) 0.011 | 1.12 (0.64-1.96) 0.686 | 1.12 (0.63-1.98) 0.703 | 1.06 (0.56-2.00) 0.855 |
|  | Mineral dust  N=2238 | L | 1.39 (0.85-2.28) 0.189 | 1.21 (0.93-1.58) 0.149 | 1.63 (1.06-2.51) 0.027 | 1.75 (1.06-2.89) 0.030 | 1.09 (0.63-1.87) 0.759 | 1.38 (0.76-2.50) 0.295 |
|  |  | H | 1.18 (0.70-1.98) 0.545 | 1.20 (0.91-1.57) 0.188 | 2.07 (1.33-3.22) 0.001 | 1.13 (0.65-1.97) 0.660 | 0.86 (0.48-1.56) 0.623 | 1.07 (0.56-2.02) 0.842 |
|  | Gases and fumes N=2911 | L | 1.20 (0.79-1.82) 0.394 | 1.13 (0.91-1.40) 0.276 | 1.16 (1.13-2.31) 0.009 | 1.58 (1.01-2.47) 0.046 | 0.85 (0.54-1.36) 0.500 | 1.24 (0.74-2.08) 0.426 |
|  |  | H | 1.31 (0.80-2.14) 0.290 | 1.16 (0.90-1.51) 0.253 | 1.82 (1.17-2.83) 0.008 | 1.19 (0.70-2.01) 0.526 | 0.90 (0.51-1.58) 0.708 | 1.17 (0.694-2.15) 0.605 |
| Pesticide group | Herbicides  N=1223 | L | 0.73 (0.27-1.87) 0.492 | 1.16 (0.75-1.79) 0.514 | 1.78 (0.90-3.50) 0.096 | 0.94 (0.39-2.25) 0.881 | 0.67 (0.24-1.88) 0.445 | 1.28 (0.52-3.16) 0.592 |
|  |  | H | 1.11 (0.55-2.23) 0.779 | 1.18 (0.82-1.69) 0.375 | 2.11 (1.21-3.66) 0.008 | 1.74 (0.91-3.34) 0.094 | 0.92 (0.42-2.02) 0.843 | 1.16 (0.53-2.53) 0.717 |
|  | Insecticides  N=1218 | L | 0.80 (0.28-2.23) 0.666 | 0.67 (0.39-1.17) 0.159 | 1.57 (0.72-3.44) 0.259 | 0.77 (0.27-2.21) 0.628 | 0.75 (0.24-2.33) 0.619 | 1.18 (0.42-3.32) 0.752 |
|  |  | H | 0.93 (0.47-1.85) 0.837 | 1.26 (0.90-1.76) 0.182 | 2.03 (1.21-3.41) 0.008 | 1.50 (0.80-2.84) 0.209 | 0.78 (0.36-1.68) 0.521 | 0.97 (0.45-2.09) 0.932 |
|  | Fungicides  N=1247 | L | 1.04 (0.42-2.53) 0.939 | 0.72 (0.43-1.23) 0.230 | 1.33 (0.59-2.97) 0.490 | 0.83 (0.31-2.25) 0.719 | 1.40 (0.57-3.46) 0.460 | 0.63 (0.19-2.07) 0.445 |
|  |  | H | 0.85 (0.43-1.69) 0.645 | 1.21 (0.87-1.69) 0.256 | 1.95 (1.17-3.27) 0.011 | 1.46 (0.77-2.75) 0.247 | 0.81 (0.38-1.72) 0.584 | 1.06 (0.50-2.23) 0.883 |
| Solvent group | Aromatic solvents N=1531 | L | 1.16 (0.65-2.07) 0.616 | 1.33 (0.99-1.79) 0.055 | 1.84 (1.14-2.98) 0.013 | 1.22 (0.69-2.24) 0.513 | 1.03 (0.56-1.92) 0.920 | 1.06 (0.53-2.13) 0.865 |
|  |  | H | 0.82 (0.22-3.10) 0.767 | 0.85 (0.42-1.73) 0.655 | 0.33 (0.04-2.64) 0.298 | 0.78 (0.20-3.06) 0.723 | 1.57 (0.49-5.05) 0.446 | 2.59 (0.86-7.77) 0.091 |
|  | Chlorinated solvents N=1331 | L | 1.12 (0.57-2.20) 0.745 | 1.25 (0.89-1.77) 0.202 | 1.03 (0.55-1.93) 0.928 | 0.93 (0.44-1.95) 0.849 | 1.18 (0.58-2.41) 0.645 | 1.27 (0.59-2.73) 0.543 |
|  |  | H | 1.72 (0.82-3.60) 0.152 | 1.53 (1.02-2.30) 0.039 | 1.36 (0.63-2.92) 0.430 | 1.06 (0.46-2.42) 0.887 | 0.85 (0.33-2.21) 0.736 | 0.85 (0.33-2.24) 0.749 |
|  | Other solvents N=1947 | L | 1.26 (0.81-1.98) 0.309 | 1.16 (0.92-1.47) 0.207 | 1.56 (1.06-2.30) 0.023 | 1.34 (0.82-2.17) 0.242 | 0.77 (0.46-1.28) 0.308 | 1.03 (0.59-1.82) 0.907 |
|  |  | H | 1.01 (0.36-2.81) 0.981 | 1.06 (0.63-1.80) 0.824 | 0.38 (0.08-1.64) 0.193 | 1.32 (0.50-3.51) 0.579 | 1.24 (0.46-3.35) 0.666 | 2.72 (1.10-6.75) 0.031 |
| Metals N=1423 | | L | 1.19 (0.58-2.43) 0.641 | 1.06 (0.74-1.53) 0.743 | 1.15 (0.58-2.27) 0.696 | 0.99 (0.47-2.09) 0.982 | 1.66 (0.79-3.48) 0.181 | 1.00 (0.43-2.32) 0.998 |
|  |  | H | 1.92 (0.90-4.07) 0.091 | 1.44 (0.96-2.15) 0.075 | 1.52 (0.71-3.27) 0.282 | 1.00 (0.44-2.29) 0.998 | 1.13 (0.44-2.93) 0.800 | 0.91 (0.35-2.40) 0.848 |

aMOR: adjusted multinomial odds ratio, adjusted for sex, education, smoking, pack-years, childhood asthma and adulthood asthma at age 53 years. L: onlylow-; H: ever-high; no exposure to any of the agents as the reference; all compared to the “non-coughers” as the reference group.

**Table S14. Adjusted associations between occupational exposures (cumulative exposure unit-year) and cough subclasses at age 53 years, compared to fully unexposed group.**

| Cumulative occupational exposures | | Cough subclasses at age 53 years | | | | | |
| --- | --- | --- | --- | --- | --- | --- | --- |
|  |  | Minimal cough (n=170) | Cough with colds only (n=991) | Cough with allergies (n=263) | Intermittent productive cough (n=174) | Chronic dry cough (n=118) | Chronic productive cough (n=127) |
|  |  | aMOR for per 10 exposure unit-year increase (95% CI), p-value | | | | | |
| Dust/gases group | Biological dust N=2403 | 1.01 (0.95-1.07) 0.688 | 0.99 (0.95-1.02) 0.379 | 1.05 (1.00-1.10) 0.034 | 1.02 (0.97-1.08) 0.499 | 1.00 (0.93-1.07) 0.979 | 1.00 (0.93-1.07) 0.907 |
|  | Mineral dust N=2218 | 1.00 (0.95-1.06) 0.954 | 1.01 (0.98-1.03) 0.690 | 1.03 (0.98-1.08) 0.186 | 0.98 (0.92-1.03) 0.395 | 0.94 (0.87-1.02) 0.128 | 0.99 (0.92-1.05) 0.698 |
|  | Gases and fumes N=2879 | 1.03 (0.99-1.08) 0.172 | 1.00 (0.98-1.03) 0.982 | 1.01 (0.96-1.05) 0.799 | 1.01 (0.96-1.05) 0.767 | 102 (0.97-1.08) 0.466 | 1.00 (0.95-1.06) 0.894 |
| Pesticide group | Herbicides N=1220 | 1.04 (0.93-1.17) 0.447 | 0.99 (0.92-1.05) 0.695 | 1.11 (1.01-1.21) 0.024 | 1.11 (1.01-1.22) 0.024 | 0.97 (0.82-1.15) 0.737 | 1.02 (0.89-1.17) 0.800 |
|  | Insecticides N=1213 | 1.01 (0.90-1.12) 0.827 | 1.00 (0.94-1.06) 0.983 | 1.12 (1.04-1.21) 0.003 | 1.07 (0.97-1.17) 0.167 | 0.94 (0.80-1.11) 0.459 | 0.97 (0.85-1.11) 0.686 |
|  | Fungicides n=1240 | 0.98 (0.88-1.10) 0.732 | 0.99 (0.94-1.05) 0.807 | 1.10 (1.02-1.18) 0.012 | 1.06 (0.97-1.15) 0.219 | 0.99 (0.87-1.12) 0.874 | 1.00 (0.89-1.13) 0.955 |
| Solvent group | Aromatic solvents n=1525 | 1.06 (0.92-1.23) 0.405 | 1.01 (0.93-1.10) 0.828 | 1.04 (0.90-1.20) 0.573 | 1.05 (0.90-1.21) 0.538 | 1.13 (0.98-1.29) 0.084 | 1.04 (0.89-1.23) 0.598 |
|  | Chlorinated solvents n=1332 | 1.05 (0.98-1.12) 0.165 | 1.03 (0.99-1.08) 0.113 | 1.03 (0.95-1.11) 0.550 | 1.01 (0.92-1.10) 0.910 | 1.00 (0.90-1.12) 0.927 | 0.95 (0.84-1.08) 0.463 |
|  | Other solvents n=1929 | 1.04 (0.95-1.15) 0.391 | 1.00 (0.94-1.06) 0.988 | 1.03 (0.94-1.13) 0.576 | 1.09 (1.00-1.19) 0.051 | 1.07 (0.96-1.19) 0.214 | 1.09 (0.98-1.21) 0.111 |
| Metals n=1419 | | 1.07 (1.01-1.14) 0.035 | 1.04 (0.99-1.08) 0.088 | 1.04 (0.96-1.12) 0.334 | 1.00 (0.92-1.09) 0.948 | 1.00 (0.90-1.11) 0.953 | 0.97 (0.86-1.08) 0.557 |

aMOR: adjusted multinomial odds ratio, adjusted for sex, education, smoking, pack-years, childhood asthma and adulthood asthma at age 53 years. No exposure (cumulative exposure unit-year=0) to any of the agents as the reference; all compared to the “non-coughers” as the reference group.

**Table S15. Adjusted associations between occupational exposures and standard cough definitions at age 53 years, compared to fully unexposed group.**

| Standard cough definitions at 53 years | | | Ever-exposure, aOR (95%CI), p-value | | | Cumulative exposure unit-year, aOR for per 10 exposure unit-year increase (95% CI), p-value | | |
| --- | --- | --- | --- | --- | --- | --- | --- | --- |
|  |  |  | Chronic cough | Chronic bronchitis | Chronic phlegm | Chronic cough | Chronic bronchitis | Chronic phlegm |
| Dust/gases group | Biological dust | L | 0.84 (0.61-1.16) 0.295 | 0.93 (0.49-1.77) 0.820 | 1.23 (0.77-1.98) 0.384 | 0.98 (0.93-1.02) 0.329 | 1.03 (0.96-1.11) 0.361 | 1.03 (0.98-1.09) 0.224 |
|  |  | H | 0.93 (0.63-1.37) 0.721 | 1.32 (0.65-2.68) 0.442 | 1.23 (0.72-2.13) 0.448 |  |  |  |
|  | Mineral dust | L | 1.01 (0.70-1.45) 0.964 | 1.48 (0.76-2.87) 0.248 | 1.37 (0.82-2.28) 0.229 | 0.99 (0.95-1.03) 0.521 | 0.99 (0.92-1.06) 0.684 | 1.00 (0.94-1.05) 0.874 |
|  |  | H | 0.81 (0.55-1.20) 0.303 | 0.82 (0.39-1.72) 0.598 | 1.00 (0.58-1.74) 0.989 |  |  |  |
|  | Gases and fumes | L | 0.91 (0.66-1.24) 0.539 | 1.15 (0.62-2.14) 0.647 | 1.37 (0.87-2.16) 0.171 | 1.01 (0.97-1.04) 0.757 | 1.00 (0.94-1.07) 0.886 | 1.01 (0.97-1.06) 0.574 |
|  |  | H | 0.82 (0.56-1.19) 0.300 | 1.06 (0.53-2.12) 0.877 | 1.24 (0.74-2.09) 0.412 |  |  |  |
| Pesticide group | Herbicides | L | 1.07 (0.60-1.90) 0.813 | 1.15 (0.41-3.22) 0.797 | 0.87 (0.37-2.04) 0.746 | 0.96 (0.87-1.06) 0.392 | 1.05 (0.91-1.21) 0.543 | 1.15 (1.05-1.26) 0.002 |
|  |  | H | 0.76 (0.45-1.25) 0.278 | 0.96 (0.39-2.37) 0.938 | 1.42 (0.75-2.70) 0.286 |  |  |  |
|  | Insecticides | L | 1.45 (0.78-2.72) 0.244 | 1.26 (0.38-4.10) 0.705 | 1.11 (0.43-2.89) 0.830 | 0.94 (0.85-1.02) 0.148 | 0.99 (0.86-1.13) 0.876 | 1.06 (0.97-1.16) 0.214 |
|  |  | H | 0.67 (0.41-1.10) 0.118 | 0.73 (0.29-1.82) 0.498 | 1.07 (0.56-2.03) 0.845 |  |  |  |
|  | Fungicides | L | 1.39 (0.75-2.56) 0.294 | 0.44 (0.09-2.10) 0.300 | 0.57 (0.19-1.76) 0.331 | 0.96 (0.89-1.04) 0.351 | 1.03 (0.91-1.16) 0.627 | 1.07 (0.99-1.17) 0.106 |
|  |  | H | 0.75 (0.46-1.21) 0.235 | 0.86 (0.36-2.05) 0.733 | 1.15 (0.61-2.16) 0.670 |  |  |  |
| Solvent group | Aromatic solvents | L | 0.84 (0.55-1.28) 0.416 | 0.81 (0.37-1.79) 0.602 | 0.83 (0.45-1.52) 0.548 | 1.06 (0.96-1.17) 0.227 | 0.89 (0.71-1.11) 0.304 | 1.01 (0.88-1.15) 0.888 |
|  |  | H | 1.79 (0.82-3.88) 0.143 | 1.90 (0.56-6.48) 0.304 | 2.18 (0.82-5.77) 0.116 |  |  |  |
|  | Chlorinated solvents | L | 1.01 (0.62-1.63) 0.980 | 1.06 (0.44-2.56) 0.896 | 1.17 (0.60-2.29) 0.650 | 0.97 (0.91-1.04) 0.436 | 0.92 (0.80-1.06) 0.267 | 0.92 (0.82-1.02) 0.115 |
|  |  | H | 0.70 (0.38-1.30) 0.258 | 0.72 (0.25-2.07) 0.545 | 0.74 (0.33-1.66) 0.462 |  |  |  |
|  | Other solvents | L | 0.79 (0.56-1.12) 0.187 | 1.08 (0.57-2.08) 0.807 | 1.24 (0.76-2.01) 0.388 | 1.04 (0.97-1.11) 0.309 | 1.02 (0.90-1.16) 0.729 | 1.08 (0.99-1.17) 0.081 |
|  |  | H | 1.38 (0.72-2.64) 0.327 | 2.37 (0.85-6.65) 0.100 | 2.62 (1.20-5.75) 0.016 |  |  |  |
| Metals | | L | 1.23 (0.74-2.04) 0.436 | 0.39 (0.26-1.72) 0.403 | 0.96 (0.47-1.96) 0.902 | 0.97 (0.91-1.04) 0.366 | 0.95 (0.84-1.07) 0.366 | 0.93 (0.84-1.02) 0.119 |
|  |  | H | 0.73 (0.39-1.36) 0.323 | 0.67 (0.24-1.86) 0.444 | 0.78 (0.35-1.74) 0.539 |  |  |  |

aOR: adjusted odds ratio, adjusted for sex, education, smoking, pack-years, childhood asthma and adulthood asthma at age 53 years. L: only-low; H: ever-high; no exposure to any of the agents as the reference. All compared to a common reference group with participants unexposed to any of the agent (i.e., no ever-exposure or cumulative exposure unit-year=0).

**Table S16A. Subgroup analysis for participants exposed to Dust/gases only, in relation to cough subclasses.**

| Dust/gases exposure only (N=1433) | | Cough subclasses at age 53 years, aMOR (95%CI), p-value | | | | | |
| --- | --- | --- | --- | --- | --- | --- | --- |
|  |  | Minimal cough | Cough with colds only | Cough with allergies | Intermittent productive cough | Chronic dry cough | Chronic productive cough |
| Biological dust | L | 0.95 (0.53-1.71) 0.861 | 1.06 (0.77-1.44) 0.732 | 1.60 (1.01-2.54) 0.046 | 1.16 (0.65-2.08) 0.616 | 0.95 (0.47-1.91) 0.878 | 1.41 (0.72-2.79) 0.316 |
|  | H | 1.39 (0.43-4.45) 0.579 | 1.23 (0.62-2.44) 0.558 | 2.64 (0.90-7.80) 0.079 | 0.98 (0.26-3.65) 0.979 | 1.76 (0.47-6.62) 0.401 | 1.43 (0.29-7.04) 0.658 |
| Cumulative exposure unit-year | | 1.04 (0.92-1.17) 0.504 | 1.01 (0.94-1.08) 0.892 | 1.08 (0.97-1.21) 0.176 | 1.00 (0.88-1.14) 0.952 | 0.95 (0.77-1.18) 0.649 | 1.00 (0.83-1.19) 0.969 |
| Mineral dust | L | 0.89 (0.48-1.65) 0.712 | 1.05 (0.76-1.45) 0.769 | 1.60 (0.97-2.65) 0.068 | 1.05 (0.57-1.91) 0.880 | 1.30 (0.65-2.59) 0.452 | 1.09 (0.52-2.29) 0.819 |
|  | H | 1.84 (0.46-7.61) 0.385 | 1.73 (0.71-4.23) 0.228 | 4.79 (1.14-20.11) 0.033 | 0.40 (0.04-3.70) 0.420 | 1.33 (0.15-11.36) 0.797 | 0.79 (0.08-8.29) 0.845 |
| Cumulative exposure unit-year | | 0.99 (0.81-1.20) 0.902 | 1.01 (0.91-1.12) 0.913 | 1.06 (0.88-1.29) 0.532 | 0.95 (0.78-1.16) 0.640 | 0.92 (0.66-1.27) 0.610 | 1.00 (0.79-1.28) 0.969 |
| Gases and fumes | L | 1.22 (0.73-2.01) 0.448 | 1.10 (0.84-1.45) 0.480 | 1.55 (1.01-2.38) 0.047 | 1.42 (0.83-2.41) 0.197 | 1.01 (0.56-1.84) 0.961 | 1.58 (0.84-2.93) 0.154 |
|  | H | 1.16 (0.36-3.72) 0.803 | 1.72 (0.95-3.13) 0.074 | 2.73 (0.99-7.49) 0.051 | 0.88 (0.26-3.01) 0.840 | 0.48 (0.06-3.81) 0.487 | 1.00 (0.19-5.17) 1.00 |
| Cumulative exposure unit-year | | 0.95 (0.82-1.10) 0.467 | 0.99 (0.93-1.06) 0.826 | 0.99 (0.87-1.13) 0.940 | 1.00 (0.89-1.12) 0.985 | 1.00 (0.86-1.17) 0.986 | 1.01 (0.87-1.16) 0.928 |
| Dust/gases composite | L | 1.15 (0.69-1.91) 0.602 | 1.01 (0.77-1.33) 0.930 | 1.46 (0.95-2.25) 0.084 | 1.36 (0.79-2.34) 0.259 | 0.72 (0.38-1.34) 0.295 | 1.25 (0.66-2.37) 0.486 |
|  | H | 1.17 (0.46-2.97) 0.734 | 1.35 (0.82-2.21) 0.239 | 2.24 (0.96-5.21) 0.031 | 1.01 (0.38-2.65) 0.987 | 1.24 (0.43-3.61) 0.688 | 1.39 (0.44-4.38) 0.574 |
| Cumulative exposure unit-year | | 1.00 (0.90-1.10) 0.952 | 0.98 (0.93-1.04) 0.543 | 1.02 (0.93-1.12) 0.717 | 0.99 (0.90-1.08) 0.770 | 0.97 (0.85-1.11) 0.702 | 1.00 (0.89-1.13) 0.969 |

**Table S16B. Subgroup analysis for participants exposed to Dust/gases only, in relation to standard cough definitions.**

| Dust/gases exposure only | | Standard cough definitions, aOR (95%CI), p-value | | |
| --- | --- | --- | --- | --- |
|  |  | Chronic cough, CC (N=1426) | Chronic bronchitis, CB (N=1429) | Chronic phlegm, CP (N=1427) |
| Biological dust | L | 1.12 (0.73-1.72) 0.604 | 1.68 (0.76-3.70) 0.198 | 1.33 (0.74-2.36) 0.339 |
|  | H | 1.49 (0.62-3.54) 0.373 | 1.79 (0.37-8.58) 0.465 | 1.11 (0.31-3.93) 0.871 |
| Cumulative exposure unit-year | | 1.00 (0.90-1.11) 0.996 | 1.01 (0.84-1.21) 0.914 | 1.00 (0.87-1.14) 0.967 |
| Mineral dust | L | 1.23 (0.79-1.90) 0.358 | 1.51 (0.67-3.41) 0.325 | 1.07 (0.57-1.98) 0.841 |
|  | H | 0.53 (0.11-2.49) 0.420 | 0.76 (0.08-7.44) 0.810 | 0.69 (0.13-3.61) 0.660 |
| Cumulative exposure unit-year | | 0.97 (0.82-1.14) 0.701 | 1.05 (0.84-1.33) 0.653 | 1.02 (0.85-1.21) 0.848 |
| Gases and fumes | L | 1.09 (0.75-1.59) 0.658 | 1.76 (0.85-3.66) 0.130 | 1.50 (0.88-2.55) 0.138 |
|  | H | 0.37 (0.11-1.26) 0.113 | 0.50 (0.06-4.37) 0.528 | 1.02 (0.31-3.32) 0.976 |
| Cumulative exposure unit-year | | 0.99 ((0.90-1.09) 0.816 | 1.01 (0.87-1.18) 0.895 | 1.02 (0.91-1.14) 0.767 |
