# Supplementary figures and images for: Associations Between Occupational Exposures and Cough Subclasses Among Middle‐Aged Australians
Source: Respirology. 2025 Apr 2;30(9):840–50. doi: 10.1111/resp.70040 (PMC12438006; doi:10.1111/resp.70040)

# Occupational risks over 10 years for different cough subclasses among middle-aged Australians

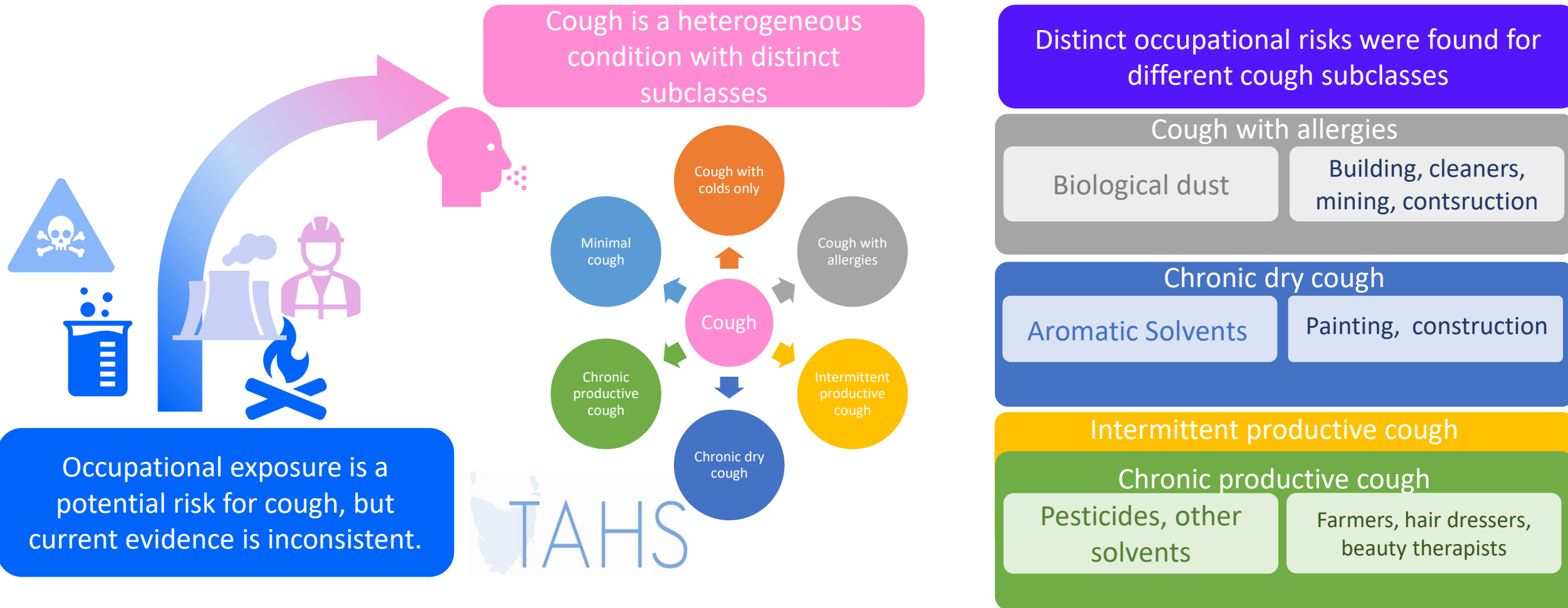

Supplement: Supplementary file 2 — Visual Abstract Occupational risks over 10 years for different cough subclasses among middle‐aged Australians [file RESP-30-840-s002.pdf]
